# β-Keto esters from ketones and ethyl chloroformate: a rapid, general, efficient synthesis of pyrazolones and their antimicrobial, *in silico* and *in vitro* cytotoxicity studies

**DOI:** 10.1186/2191-2858-3-6

**Published:** 2013-07-19

**Authors:** Ramasamy Venkat Ragavan, Kalavathi Murugan Kumar, Vijayaparthasarathi Vijayakumar, Sundaramoorthy Sarveswari, Sudha Ramaiah, Anand Anbarasu, Sivashanmugam Karthikeyan, Periyasamy Giridharan, Nalilu Suchetha Kumari

**Affiliations:** 1 Centre for Organic and Medicinal Chemistry, VIT University, Vellore 632 014, India; 2 Medical and Biological Computing Laboratory, School of Biosciences and Technology, VIT University, Vellore 632 014, India; 3 Industrial Biotechnology Division, School of Bio Sciences and Technology, VIT University, Vellore 632 014, India; 4 Department of Oncology, HCS & HTS, Piramal Life Sciences Ltd. Guregaon (E), Mumbai 400063, India; 5 Department of Biochemistry, K.S. Hegde Medical Academy, Deralakatte 574 162, India

**Keywords:** β-keto esters, Ethyl chloroformate, Pyrazolones, Efficient synthesis, Anti-bacterial activity, Fungicidal activity, Cytotoxicity studies

## Abstract

**Background:**

Pyrazolones are traditionally synthesized by the reaction of β-keto esters with hydrazine and its derivatives. There are methods to synthesize β-keto esters from esters and aldehydes, but these methods have main limitation in varying the substituents. Often, there are a number of methods such as acylation of enolates in which a chelating effect has been employed to lock the enolate anion using lithium and magnesium salts; however, these methods suffer from inconsistent yields in the case of aliphatic acylation. There are methods to synthesize β-keto esters from ketones like caboxylation of ketone enolates using carbon dioxide and carbon monoxide sources in the presence of palladium or transition metal catalysts. Currently, the most general and simple method to synthesize β-keto ester is the reaction of dimethyl or ethyl carbonate with ketone in the presence of strong bases which also requires long reaction time, use of excessive amount of reagent and inconsistent yield. These factors lead us to develop a simple method to synthesize β-keto esters by changing the base and reagent.

**Results:**

A series of β-keto esters were synthesized from ketones and ethyl chloroformate in the presence of base which in turn are converted to pyrazolones and then subjected to cytotoxicity studies towards various cancer cell lines and antimicrobial activity studies towards various bacterial and fungal strains.

**Conclusion:**

The β-keto esters from ethyl chloroformate was successfully attempted, and the developed method is simple, fast and applicable to the ketones having the alkyl halogens, protecting groups like Boc and Cbz that were tolerated and proved to be useful in the synthesis of fused bicyclic and tricyclic pyrazolones efficiently using cyclic ketones. Since this method is successful for different ketones, it can be useful for the synthesis of pharmaceutically important pyrazolones also. The synthesized pyrazolones were subjected to antimicrobial, docking and cytotoxicity assay against ACHN (human renal cell carcinoma), Panc-1 (human pancreatic adenocarcinoma) and HCT-116 (human colon cancer) cell line, and lead molecules have been identified. Some of the compounds are found to have promising activity against different bacterial and fungal strains tested.

## Background

Pyrazolones are important class of heterocyclic ring systems that have been used extensively in pharmaceutical industry [1,2] due to their numerous applications as analgesic, antipyretic, antiarthritic, uricosuric, anti-inflammatory and antiphlogistic properties. Especially, a pyrazolone derivative (edaravone) [3] acts as a radical scavenger to interrupt the peroxidative chain reactions and membrane disintegrations associated with ischemia [4-6]. Some of the aryloxypyrazolone derivatives are useful in the treatment of a variety of disorders caused by human immunodeficiency virus and other genetic ailments caused by retroviruses such as acquired immune deficiency syndrome [7]. In addition, these compounds are appropriate precursors for industrial preparation of herbicides [8], liquid crystals [9,10], dyes [11], thermally stable polymers [12] and colour photographical compounds [13]. Azadienophiles from the chemical oxidation of pyrazolones are acting as suitable substrates for hetero Diels-Alder reactions [14].

Pyrazolones are traditionally synthesized by the reaction of β-keto esters with hydrazine and its derivatives [15-21]. There are a number of alternative methods to synthesize pyrazolones which are documented in the literature [22-33] but tend to have serious drawbacks such as step-intensive, carbon monoxide usage and sensitive palladium catalysts. These factors revealed that using β-keto esters as an intermediate is the broadest and most efficient way to synthesize pyrazolones. There are methods to synthesize β-keto esters from esters [34-37] (Claisen condensation) and aldehydes [38,39], but these methods have main limitation in varying the substituents. Often, a number of methods such as acylation of enolates of malonates [40,41], acylation of Meldrum's acid [42-45], mixed malonate esters [46,47] and bistrimethylsilylmalonate [48,49] have a chelating effect employed to lock the enolate anion of malonate using lithium and magnesium salts [50,51]; however, these methods suffer from inconsistent yields in the case of aliphatic acylation. There are methods to synthesize β-keto esters from ketones like caboxylation of ketone enolates [52-54] using carbon dioxide and carbon monoxide sources in the presence of palladium or transition metal catalysts. Currently, the most general and simple method to synthesize β-keto ester is the reaction of dimethyl or ethyl carbonate with ketone in the presence of strong bases [55,56]. This method requires long reaction time, use of excessive amount of reagent and inconsistent yield. These factors lead us to develop a simple method to synthesize β-keto esters by changing the base and reagent.

## Methods

### Antibacterial study

The newly synthesized pyrazoles for their antibacterial activity against *Escherichia coli* (ATTC-25922), *Staphylococcus aureus* (ATTC-25923), *Pseudomonas aeruginosa* (ATTC-27853) and *Klebsiella pneumonia* (recultured) bacterial strains by the disc diffusion method [57,58]. The discs measuring 6.25 mm in diameter were punched from Whatman No. 1 filter paper (GE Healthcare, Little Chalfont, UK). Batches of 100 discs were dispensed to each screw-capped bottle and sterilized by dry heat at 140°C for an hour. The test compounds were prepared with different concentrations using DMF. One milliliter containing 100 times the amount of chemical in each disc was added to each bottle, which contains 100 discs. The discs of each concentration were placed in triplicate in a nutrient agar medium separately seeded with fresh bacteria. The incubation was carried out at 37°C for 24 h. Solvent and growth controls were kept, and the zones of inhibition and minimum inhibitory concentrations (MIC) were noted. Results of these studies were given in Table 1 and compared with the standard ciprofloxacin.

**Table 1:** Antibacterial activity of the newly synthesized compounds

	***S. aureus***	***E. coli***	***P. aeruginosa***	***K. pneumonia***
**Compound number**				
**1**	21 (6.25)	17 (6.25)	18 (6.25)	20 (6.25)
**2**	20 (6.25)	18 (6.25)	19 (6.25)	21 (6.25)
**3**	23 (6.25)	19 (6.25)	20 (6.25)	22 (6.25)
**4**	20 (6.25)	17 (6.25)	18 (6.25)	19 (6.25)
**5**	16 (100)	17 (100)	12 (100)	14 (100)
**6**	17 (100)	17 (100)	11 (100)	15 (100)
**7**	26 (12.5)	23 (12.5)	21 (12.5)	20 (12.5)
**8**	19 (100)	23 (100)	22 (100)	16 (100)
**9**	26 (6.25)	23 (6.25)	21 (6.25)	20 (6.25)
**10**	22 (6.25)	18 (6.25)	19 (6.25)	21 (6.25)
**11**	17 (6.25)	21 (6.25)	20 (6.25)	21 (6.25)
**13**	28 (12.5)	22 (12.5)	25 (12.5)	23 (12.5)
**14**	29 (12.5)	25 (12.5)	22 (12.5)	21 (12.5)
**15**	23 (6.25)	20 (6.25)	21 (6.25)	22 (6.25)
**17**	31 (12.5)	25 (12.5)	27 (12.5)	20 (12.5)
**19**	18 (6.25)	19 (6.25)	22 (6.25)	20 (6.25)
**20**	24 (6.25)	25 (6.25)	26 (6.25)	26 (6.25)
**21**	30 (12.5)	24 (12.5)	25 (12.5)	22 (12.5)
**23**	24 (12.5)	27 (12.5)	24 (12.5)	23 (12.5)
**24**	16 (100)	17 (100)	12 (100)	14 (100)
**25**	21 (12.5)	24 (12.5)	26 (12.5)	22 (12.5)
**26**	21 (6.25)	23 (6.25)	22 (6.25)	20 (6.25)
Ciprofloxacin	23 (6.25)	32 (6.25)	28 (6.25)	24 (6.25)

Zone of inhibition (mm); MIC (μg/mL) given in parenthesis.

### Antifungal activity

Newly synthesized pyrazoles were screened for their antifungal activity against *Aspergillus flavus* (NCIM no. 524), *Aspergillus fumigates* (NCIM no. 902), *Penicillium marneffei* (recultured) and *Trichophyton mentagrophytes* (recultured) in dimethylsulfoxide (DMSO) by the serial plate dilution method [34-36]. Sabouraud agar media was prepared by dissolving peptone (1 g), d-glucose (4 g) and agar (2 g) in distilled water (100 mL), and the pH was adjusted to 5.7. Normal saline was used to make a suspension of spores of fungal strain for lawning. A loopful of particular fungal strain was transferred to 3 mL of saline to get a suspension of corresponding species. Agar media of 20 mL was poured into each Petri dish. An excess of suspension was decanted, and the plates were dried by placing them in an incubator at 37°C for 1 h. Using an agar, punch wells were made on these seeded agar plates, and 10 to 50 μg/mL of the test compounds in DMSO were added into each labelled well. A control was also prepared for plates in the same way using solvent DMSO. The Petri dishes were prepared in triplicate and maintained at 37°C for 3 to 4 days. Antifungal activity was determined by measuring the inhibition zone. The results of these studies were given in Table 2 and compared with the standard ciclopiroxolamine.

**Table 2:** Antifungal activities of the newly synthesized compounds

	***Trichophyton***	***Penicillium***	***A. flavus***	***A. fumigates***
**Compound number**				
**1**	25 (6.25)	23 (6.25)	26 (6.25)	27 (6.25)
**2**	24 (6.25)	25 (6.25)	24 (6.25)	26 (6.25)
**3**	29 (6.25)	26 (6.25)	27 (6.25)	28 (6.25)
**4**	21 (6.25)	22 (6.25)	26 (6.25)	22 (6.25)
**5**	16 (12.5)	17 (12.5)	12 (12.5)	14 (12.5)
**6**	17 (12.5)	17 (12.5)	11 (12.5)	15 (12.5)
**7**	24 (12.5)	21 (12.5)	21 (12.5)	20 (12.5)
**8**	26 (12.5)	24 (12.5)	27 (12.5)	23 (12.5)
**9**	27 (12.5)	25 (12.5)	28 (12.5)	22 (12.5)
**10**	20 (6.25)	22 (6.25)	17 (6.25)	22 (6.25)
**11**	21 (6.25)	21 (6.25)	23 (6.25)	21 (6.25)
**13**	22 (12.5)	25 (12.5)	27 (12.5)	23 (12.5)
**14**	30 (12.5)	22 (12.5)	26 (12.5)	24 (12.5)
**15**	26 (12.5)	23 (12.5)	27 (12.5)	23 (12.5)
**17**	31 (12.5)	25 (12.5)	28 (12.5)	23 (12.5)
**19**	25 (6.25)	24 (6.25)	27 (6.25)	24 (6.25)
**20**	28 (12.5)	29 (12.5)	25 (12.5)	25 (12.5)
**21**	31 (12.5)	28 (12.5)	27 (12.5)	24 (12.5)
**23**	29 (12.5)	27 (12.5)	26 (12.5)	21 (12.5)
**24**	23 (12.5)	26 (12.5)	23 (12.5)	25 (12.5)
**25**	21 (6.25)	20 (6.25)	21 (6.25)	23 (6.25)
**26**	25 (12.5)	22 (12.5)	27 (12.5)	28 (12.5)
Standard	27 (3.125)	23 (6.25)	27 (3.125)	26 (6.25)

Zone of inhibition (mm), MIC (μg/mL) given in parenthesis and ciclopiroxolamine as standard.

### Docking studies

All the synthesized compounds **1** to **26** have been subjected to the docking studies against ACHN (human renal cell carcinoma), Panc-1 (human pancreatic adenocarcinoma) and HCT-116 (human colon cancer) and then subjected to WST-1 cytotoxicity assay. Based on the crystal structures of the target proteins and high-throughput molecular docking methods, four phases of Gemdock methods were used. These phases include target protein structure analysis, ligand optimization, molecular docking and post-docking analysis. The macro- and small-molecule optimization phase involved in editing the structural coordinates of the target protein and compounds. The third phase was molecular docking method to identify potential leads for the target protein; then, the fourth phase was post-docking analysis to identify best conformation of ligand molecule. In the present study, the coordinates of three cancer target proteins were selected and obtained from the Protein Data Bank (PDB) [59]. The PDB entry 1SVC (pancreatic cancer), 3B8Q (renal cancer) and 4FLH (colon cancer) were selected for structural analysis based on its high-resolution crystallographic structure. For docking studies, the PDB coordinates of obtained target proteins were edited by removing the co-crystallized ligand molecule. The crystallographic water molecules were eliminated from the atomic coordinate file, and the polar hydrogen atoms and Kollman united charges were added to each target protein, followed by structure optimization and refinement using spdbv viewer [60]. The synthesized chemical compound structures were sketched with the help of ChemSketch [61]. A three-dimensional (3D) conversion and geometry optimization of all the compounds were performed using chimera [62] for flexible conformations of the compounds during the docking. To study the detailed intermolecular interactions between the target protein and the ligand molecule, automated docking program iGEMDOCK (a generic evolutionary method for molecular DOCKing) software was used [63]. iGEMDOCK integrated the virtual screening, molecular docking, post-screening analysis and visualization steps. We selected nuclear factor kappa b (NF-κb), vascular endothelial growth factor receptor-2 and human phosphoinositide 3-kinase (PI3K-gamma) (PDB ID: 1SVC, 3B8Q and 4FLH, respectively) as target proteins to carry out the docking analysis of our synthesized compounds. The 3D coordinates of each therapeutic target protein were implemented through the GEMDOCK graphical environment interface. Then, the default option was used to import the 3D coordinates of 27 synthesized compounds. Before docking, the output path was set. GEMDOCK default parameters included the population size (*n* = 200), generation (*g* = 70) and number of solutions (*s* = 10) to compute the probable binding conformation of synthesized compounds. Then, the docking run was started using GEMDOCK scoring function. After docking, the individual binding conformation of each ligand was observed, and their binding affinity with the target proteins was analyzed. The best binding pose and binding energy of each ligand was selected. In the post-docking analysis, van der Waals score, *Z* score and the details of interacted residues were saved in output folder. Protein-ligand binding site was analyzed and visualized using PyMOL [64]. The three-dimensional structures of NF-κb, vascular endothelial growth factor receptor-2 and human phosphoinositide 3-kinase are analyzed, and synthesized compounds **1** to **26** are optimized to have minimal potential energy using chimera. After minimization, all the ligands are docked into each target protein to study the molecular basis of interaction and binding affinity of all the synthesized compounds. From the docking analysis, we listed best conformers based on total energy, *Z* score and van der Waals score (VDW) for each ligand molecule (Tables 3,4,5). The best docking poses for each ligand molecule into each target protein are determined, and the one having the lowest binding energy among the 20 different poses generated. The lower energy scores represent better protein-ligand binding affinity compared to higher energy values.

**Table 3:** Docking results of synthesized compounds in the binding site of nuclear factor kappa b

**Compound number**	**Total energy**	***Z *score**	**VDW**
**1**	−74.15	−73.1	−73.15
**2**	−66.2304	−70.6	−56.7448
**3**	−78.2994	−90.8	−65.8385
**4**	−42.783	−45.93	−68.7026
**5**	−50.5602	−54.9	−50.366
**6**	−88.1508	−110.2	−70.312
**7**	−32.2859	−40.6	−55.3665
**8**	−49.5672	−50.8	−56.3479
**9**	−62.3895	−62.4	−50.3603
**10**	−74.4438	−72.3	−70.4519
**11**	−90.4298	−117.4	−80.5608
**12**	−83.3089	−90.4	−67.7796
**13**	−42.6816	−50.3	−60.7439
**14**	−91.9971	−119.9	−74.1695
**15**	−35.7564	−46.7	−68.4413
**16**	−72.932	−69.9	−60.4764
**17**	−60.4516	−60.2	−60.3893
**18**	−34.3128	−101.7	−53.5055
**19**	−41.0148	−50.9	−63.3827
**20**	−35.2375	−40.6	−87.3575
**21**	−79.2554	−85.2	−51.5586
**22**	−39.9575	−42.3	−67.6976
**23**	−32.1991	−42.3	−63.8354
**24**	−58.4277	−60.9	−67.0823
**25**	−58.424	−60.9	−53.7606
**26**	−30.0129	−44.6	−44.8782

**Table 4:** Docking results of synthesized compounds in the binding site of vascular endothelial growth factor receptor-2

**Compound number**	**Total energy**	***Z *score**	**VDW**
**1**	−75.0934	−72.2	−79.4166
**2**	−78.2062	−75.1	−69.6532
**3**	−70.5653	−95.6	−68.46
**4**	−78.7892	−72.3	−63.191
**5**	−65.564	−78.9	−59.3404
**6**	−86.6543	−105.7	−80.5888
**7**	−71.8927	−63.17	−59.0905
**8**	−95.9923	−120.5	−94.7849
**9**	−79.948	−71.9	−56.0692
**10**	−73.5766	−80	−86.6021
**11**	−72.3245	−73.6	−73.1902
**12**	−75.4277	−72.2	−70.7142
**13**	−85.3265	−94.6	−54.4274
**14**	−75.329	−75.3	−71.2839
**15**	−80.914	−75.1	−73.6739
**16**	−75.3033	−91.5	−63.0176
**17**	−68.7853	−74.3	−69.6841
**18**	−104.9856	−125.5	−105.697
**19**	−92.6464	−115.2	−87.8944
**20**	−74.3443	76.7	−70.902
**21**	−62.3597	−73.3	−50.6291
**22**	−60.2348	−78.2	−65.3015
**23**	−77.191	−75.6	−63.8723
**24**	−82.723	−77.2	−68.4238
**25**	−80.73	−75	−56.4072
**26**	−75.093	−104.3	−48.6469

**Table 5:** Docking results of synthesized compounds in the binding site of phosphoinositide 3-kinase

**Compound number**	**Total energy**	***Z *score**	**VDW**
**1**	−119.541	−122.5	−78.0144
**2**	−67.4663	68.3	−53.8734
**3**	−105.3452	−90.9	−68.1224
**4**	−75.0481	−75	−70.3258
**5**	−77.1818	−77.2	−61.47
**6**	−101.23	−105.1	−55.6405
**7**	−96.8291	−110.9	−54.3328
**8**	−92.0488	−92	−61.893
**9**	−75.3184	−75.3	−62.0764
**10**	−119.421	−120.5	−76.7195
**11**	92.8443	−92.3	−62.324
**12**	−83.9072	−83.9	−85.1019
**13**	−80.5887	−80.6	−66.5004
**14**	−107.157	−102.2	−62.1177
**15**	−76.9716	−77	−70.2072
**16**	−94.8943	−106.4	−52.3224
**17**	−90.9786	−91.4	−66.5817
**18**	−110.067	−91	−80.4918
**19**	−83.2508	−83.3	−54.2574
**20**	−76.3532	−76.3	−86.3532
**21**	−82.2975	−82.3	−54.9572
**22**	−74.2083	−74.2	−71.0281
**23**	−81.0895	−81.1	−63.5472
**24**	−76.2358	−76.2	−58.7925
**25**	−67.4663	−67.5	−49.4389
**26**	−80.9917	−81.1	−48.6582

### Cytotoxicity studies

The compounds **1** to **26** have been subjected to cyctotoxicity studies. Towards this, a panel of three cancer cells representing multiple cancers of clinical relevance were obtained from American Type Culture Collection (ATCC), namely ACHN (human renal cell carcinoma), Panc-1 (human pancreatic adenocarcinoma) and HCT-116 (human colon cancer). Cells were maintained in Dulbecco's modified Eagle's medium (DMEM) medium containing 10% heat-inactivated fetal bovine serum and kept in humidified 5% CO_2_ incubator at 37°C. Logarithmically, growing cells were plated at a density of 5 × 10^3^ cells/well in a 96-well tissue culture grade micro-plate and allowed to recover overnight. The cells were challenged with varying concentrations of compounds for 48 h. Control cells received standard media containing dimethylsulfoxide vehicle at a concentration of 0.2%. After 48 h of incubation, cell toxicity was determined by the Cell Counting Kit-8 (CCK-8) reagent (Dojindo Molecular Technologies, Inc., Kumamoto, Japan); (WST-1 [2-(2-methoxy-4-nitrophenyl)-3-(4-nitrophenyl)-5-(2,4-disulfophenyl)]-2*H*-tetrazolium, monosodium salt assay). In accordance with the manufacturer's instructions [36], 5 μL/well CCK-8 reagent was added, and plates were incubated for 2 h. Cytotoxicity of all the compounds have been determined by measuring the absorbance on Tecan Sapphire multi-fluorescence micro-plate reader (Tecan GmbH, Crailsheim, Germany) at a wavelength of 450 nm corrected to 650 nm and normalized to controls. Each independent experiment was performed thrice and tabulated in Table 6.

**Table 6:** Cytotoxic activity of the newly synthesized compounds 1 to 26

	**Concentration (μg/mL)**	**Percentage of cytotoxicity/anti-proliferation**		
		**Panc1 (pancreas)**	**ACHN (renal)**	**HCT116 (colon)**
**Compound number**				
**1**	10	−75	−7	−138
**2**	10	−64	5	1
**3**	10	−78	0	−16
**4**	10	−10	20	−12
**5**	10	−20	−3	6
**6**	10	−101	−25	−116
**7**	10	14	−16	−115
**8**	10	−15	−31	−107
**9**	10	−56	19	8
**10**	10	−75	−7	−138
**11**	10	−117	7	−107
**12**	10	−89	13	−70
**13**	10	−14	3	5
**14**	10	−118	−19	−123
**15**	10	12	5	17
**16**	10	−71	−10	−112
**17**	10	−51	4	−102
**18**	10	4	−41	−128
**19**	10	−10	−26	−80
**20**	10	71	73	79
**21**	10	−80	−5	−20
**23**	10	12	−7	−6
**24**	10	6	2	−103
**25**	10	−45	−18	−64
**26**	10	−7	−12	1
Tannase	10	17.3	12.4	9.7

## Results and discussion

**Scheme 1:**
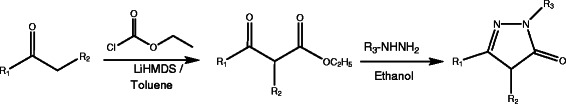
Synthesis of β-keto esters from ethyl chloroformate and its conversion into pyrazolones.

In continuation of our interest towards the synthesis of β-keto esters and pyrazolones [65-67], we made an attempt to synthesize β-keto esters from ethyl chloroformate in the presence of base which in turn are converted into pyrazolones *in situ* by the addition of either hydrazine or its derivatives, since we hypothesized that an enolate may react cleanly with highly electrophilic ethyl chloroformate to give β-keto esters. We tested our hypothesis in the synthesis of representative compound **12** by varying the solvents as well as bases (Scheme 1). The effects of base and solvent on the yield of **12** have been summarized and are given in Table 7.

**Table 7:** Effect of solvent and base on the yield of 12

**Base**	**Ketone (eq.)**	**Solvent**	**Temperature**	**Yield (%)**^**a**^
LiHMDS (1.0M THF) (1 eq.)	3	THF	−78°C	68
LiHMDS (1.0M THF) (2 eq.)	3	Toluene	−78°C	56
KHMDS (3 eq.)	7	Toluene	−78°C	42
NaH (2 eq.)	50	THF	−78°C	17
NaOMe (2 eq.)	75	THF	Reflux	0
KOtBu (3 eq.)	10	THF	25°C	19
LiHMDS (1.0M THF) (3.5 eq.)	7	Toluene	−50°C to−30°C	92

^a^Isolated yield.

The formation of β-keto ester was found to be in better yield when LiHMDS was used as a base. When other bases are used, the formation of β-keto ester intermediate from ketones was very slow, and the reactions were also found to be incomplete even after 4 to 5 h of stirring at r.t. The addition of hydrazine hydrate to the latter reaction mixtures gave the desired product in low yield (Table 8), and the corresponding hydrazone of ketones was isolated as the major product. After finding the suitable base, the reaction conditions were optimized further by varying the solvents to improve the yield. It was found that the hydrocarbon solvent (toluene) produced better yield compared to the cyclic ether solvent (THF). This may be due to the possible destabilization of formed intermediate with charge in the case of hydrocarbon solvent like toluene, and hence, the formed enolate reacts with ethyl chloroformate smoothly.

**Table 8:** Synthesis of β-keto esters by cross-Claisen condensation

**Compound number**	**Ester**	**Product**	**Yield (%)**
**1**			78
**2**			75
**3**			64
**4**			72
**5**			80
**6**			69
**7**			74
**8**			74
**9**			58
**10**			70
**11**			80
**12**			92
**13**			72
**14**			77
**15**			84
**16**			78
**17**			65^a^
**18**			61
**19**			48
**20**			74
**21**			74
**22**			0^a^
**23**			81
**24**			78
**25**			81
**26**			79
**27**			0^a^

^a^Percentage of products in crude LC-MS.

After optimizing the reaction with the suitable base (LiHMDS) and solvent (toluene), the same conditions were employed for the synthesis of various β-keto esters which in turn are converted into their corresponding pyrazolones **1** to **21** and **23** to **26***in situ* by the addition of either hydrazine or its derivatives to prove the generality of the reaction, and the results are tabulated in Table 8. The reactions have been monitored by thin layer chromatography (TLC), and the obtained crude products were purified by column chromatography. The β-keto esters were efficiently converted into their corresponding pyrazolones with good to excellent yields. All the synthesized compounds **1** to **21** and **23** to **26** have been characterized through IR, ^1^H NMR, ^13^C NMR and mass spectral data. The examination of the ^1^H NMR spectrum of **26** clearly shows that the formation of doublet at *δ* 1.34 ppm with the coupling constant of 6.92 Hz integrating for six protons is due to the two methyl groups of isopropyl substituent at C3 of pyrazolone moiety. A multiplet between *δ* 2.79 and 2.49 ppm integrating for one proton is due to the methine proton of *iso*-propyl substituent at C3 of pyrazolone moiety. The singlet at *δ* 3.33 ppm integrating for one proton is due to the proton at C4. Two broad singlets that appeared between *δ* 9.5 to 9.3 ppm and *δ* 11.2 to 11.1 ppm integrating for one proton each are due to -NH and -OH protons, respectively. This supports the ^1^H NMR findings that pyrazolone moiety is in its enol rather than the keto form since the spectrum was recorded in deuterated DMSO solvent. Similarly, the examination of the ^13^C NMR spectrum reveals the following points. The two signals that appeared at aliphatic regions 22.24 and 25.69 ppm are due to methyl and methine carbon, respectively, of the *iso*-propyl substituent at C3 of the pyrazolone moiety. The signal at 86.22 ppm is due to the C4. The two downfield signals appeared at 160.75 and 150.39 ppm. The relatively downfield signal has been assigned as C5, and the relatively upfield has been assigned as C3. The *m*/*z* observed at 126.9 in liquid chromatography-mass spectrometry (LC-MS) spectrum also supports the formation of compound **26**. In the similar way, the chemical shifts of all the other compounds have been assigned and are included in the experimental part. Some of the compounds **4**, **7**, **16**, **21**, **23** and **24** have been crystallized and subjected to the single crystal X-ray diffraction studies [68-75] and are available in the literature (Ortep plots are included in the Additional file 1); particularly, sample **4** has been crystallized as both in keto form and enol form. All the above discussions clearly revealed the formation of the desired products. This method is very simple, fast and applicable to the ketones having the alkyl halogens, protecting groups like Boc and Cbz that were tolerated and proved to be useful in the synthesis of fused bicyclic and tricyclic pyrazolones efficiently using cyclic ketones. Since this method is successful for different ketones, it can also be useful for the synthesis of pharmaceutically important pyrazolones.

We have investigated the newly synthesized pyrazoles for their antibacterial activity against *E. coli* (ATTC-25922), *S. aureus* (ATTC-25923), *P. aeruginosa* (ATTC-27853) and *K. pneumonia* (recultured) bacterial strains by the disc diffusion method [57,58]. Results of these studies were given in Table 1 and compared with the standard ciprofloxacin. Most of the synthesized compounds exhibited very good bacterial activity; particularly, compounds **7**, **13**, **14**, **23**, **25** and **26** have shown very good inhibition against all the bacterial strains tested. Compounds **9** to **11**, **13**, **14**, **19**, **20** and **26** have shown a moderate to good inhibition against all the bacterial strains. Compounds **8** and **24** have poor bacterial activity. The SAR studies on these compounds revealed that the aliphatic substituents (either cyclic or acyclic) on the main cage increase their biological activities. On the other hand, compounds bearing the aromatic substituents and the fused ring systems decrease their activity. Halogen substitution in alkyl group also reduces their activity. Some of the tested compounds are equipotent or more potent than the standards used.

Newly synthesized pyrazoles were screened for their antifungal activity against *A. flavus* (NCIM no. 524), *A. fumigates* (NCIM no. 902), *P. marneffei* (recultured) and *T. mentagrophytes* (recultured) in DMSO by the serial plate dilution method [34-36]. Most of the tested compounds exhibited good fungicidal activities; particularly, compounds **10**, **11**, **19** and **25** were found to be highly potent to all the four fungi tested. Compounds **1** to **9**, **13** to **15**, **17**, **20**, **21**, **23**, **25** and **26** were shown to have good to moderate activity to all the fungi tested.

**Figure 1:**
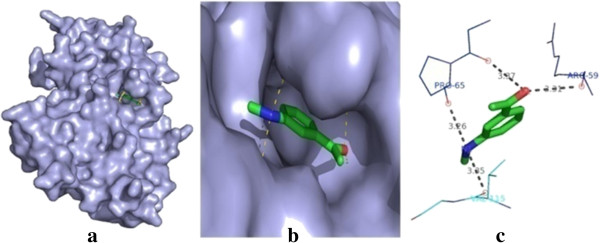
**Molecular docking result of compound 14. (a)** The docked poses of compound **14** at the site of nuclear factor kappa b; target protein is shown in the surface model, and the ligand is shown in the stick model. **(b)** A close-up view of the docked pose of compound **14**. **(c)** The amino acid residue interaction, hydrogen bond networks in the binding pocket and the distance (in Angstrom units) of bonds are shown.

All the synthesized compounds **1** to **26** have been subjected to the docking studies against ACHN (human renal cell carcinoma), Panc-1 (human pancreatic adenocarcinoma) and HCT-116 (human colon cancer) and then subjected to WST-1 cytotoxicity assay. Among the 26 synthesized compounds, compounds **14**, **20** and **4** are found to have least binding energy value and *Z* score value. These compounds are more stable ligand-receptor complex amongst other compounds. Compound **14** shows the best binding conformation with nf-κb (total energy = −91.9971 kcal/mol, *Z* score = −119). The best binding mode of compound **14** at the NF-κb binding site and the residues involved in the interaction, corresponding two-dimensional (2D) interaction models, hydrogen bonds and bond distance are shown in Figure 1. Compound **14** binds to the binding sites and forms three hydrogen bonds with NF-κb involved in pancreatic cancer. It can be seen in Figure 1 that nitrogen atoms of compound **14** formed a hydrogen bond with Pro-65 and Val115. In addition, Arg59 has one H bond with the bond distance of 3.91 Å. The binding pose and interaction mode of compound **14** are shown in Figure 1.

**Figure 2:**
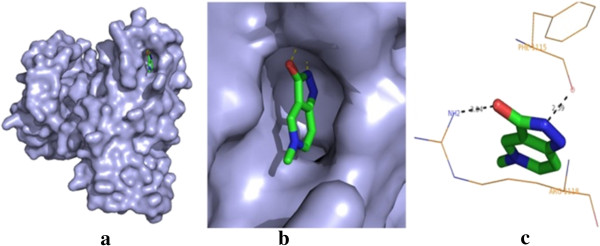
**Molecular docking result of compound 18. (a)** Binding pose of compound **18** in the vascular endothelial growth factor receptor-2. **(b)** A close-up view of the binding pose of compound **18**; protein structure is shown in the surface model, and the ligand is shown in the stick model. **(c)** H bond networks with protein residues are shown.

**Figure 3:**
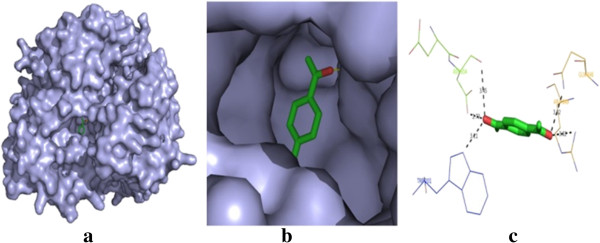
**Molecular docking result of compound 1. (a)** Docked poses of compound **1** in human phosphoinositide 3-kinase binding site. **(b)** A close-up view of the docked pose of compound **1**; protein structure is shown in the surface model, and the ligand is shown in the stick model (color by atom). **(c)** H bond networks and bond distance are shown.

The post-docking analysis of compound **18** has shown higher affinity with VGFR2 which has key role in renal cancer development (total energy = −104.9856 kcal/mol, *Z* score = −125.5). Compound **18** binds to the VGFR2 and forms one H bond interaction with Arg118 and Phe115 residues. The best binding pose of compound **18** in the VGFR2 and corresponding 2D interaction models, hydrogen bonds and bond distance are depicted in Figure 2. Docking analysis of compound **1** has shown the best conformation with PI3K (total energy = −119.541 kcal/mol, *Z* score = −125.5). The binding affinity of compound **1** towards PI3K is investigated in detail. On analysis of the interaction and position of compound **1** in the PI3K binding site, it is observed that five H bonds are found, and the amino acid residues Asp654, Gln846, ARG649 and Trp201 participated in the interaction. The surface of PI3K with compound **1** along with the main contact residues of PI3K is labelled, and hydrogen bond distances are shown in Figure 3.

In continuation of the docking analysis, the compounds **1** to **26** have been subjected to the cyctotoxicity studies. Towards this, a panel of three cancer cells representing multiple cancers of clinical relevance were obtained from ATCC, namely ACHN (human renal cell carcinoma), Panc-1 (human pancreatic adenocarcinoma) and HCT-116 (human colon cancer). Cells were maintained in DMEM containing 10% heat-inactivated fetal bovine serum and kept in humidified 5% CO_2_ incubator at 37°C. Logarithmically growing cells were plated at a density of 5 × 10^3^ cells/well in a 96-well tissue culture grade micro-plate and allowed to recover overnight. The cells were challenged with varying concentration of compounds for 48 h. Control cells received standard media containing dimethylsulfoxide vehicle at a concentration of 0.2%. After 48 h of incubation, cell toxicity was determined by the CCK-8 reagent (Dojindo Molecular Technologies, Inc.); (WST-1 [2-(2-methoxy-4-nitrophenyl)-3-(4-nitrophenyl)-5-(2,4-disulfophenyl)]-2*H*-tetrazolium, monosodium salt assay). In accordance with the manufacturer's instructions [36], 5 μL/well CCK-8 reagent was added, and plates were incubated for 2 h. Cytotoxicity of all the compounds have been determined by measuring the absorbance on Tecan Sapphire multi-fluorescence micro-plate reader (Tecan GmbH, Germany) at a wavelength of 450 nm corrected to 650 nm and normalized to controls. Each independent experiment was performed thrice and tabulated in Table 6. The compound **18** was found to be inhibitive against only ACHN (human renal cell carcinoma) cell lines. The compounds **1** and **10** were found to be inhibitive against HCT-116 (human colon cancer) cell lines. The compound **14** was found to be inhibitive against Panc-1 (human pancreatic adenocarcinoma) as well as HCT-116 (human colon cancer) cell lines. The docking poses of the compounds **1**, **10**, **14** and **18** reveals that these molecules are having either more or strong hydrogen bonding interactions with the target molecules which may be due to the presence of either *O*-alkyl or *O*-aryl or cyanide groups in it, and hence, these molecules are found to have better activity.

## Experimental

### General

All the NMR spectra were recorded using Bruker AMX 400 or Bruker DPX 300 instrument (Billerica, MA, USA) with 5-mm PABBO BB-1H tubes. ^1^H NMR spectra were recorded using approximately 0.03 M solutions in *d*_6_-DMSO at 300 or 400 MHz with tetramethylsilane (TMS) as internal reference. ^13^C NMR spectra were recorded using approximately 0.05 M solutions in *d*_6_-DMSO at 75 or 100 MHz with TMS as internal reference. In many cases, pyrazolones were recorded in the enol form, whenever *d*_6_-DMSO was used as solvent. Melting points were determined by Buchi B-545 apparatus (Golden Valley, MN, USA). LC-MS were obtained using Agilent 1200 series LC (Santa Clara, CA, USA) and MicromasszQ spectrometer (Manchester, UK).

All reagents were purchased from Sigma-Aldrich (St. Louis, MO, USA) and used as received. LiHMDS solutions were kept under nitrogen atmosphere after opening. Dry toluene, AcOH and EtOH were supplied by Spectrochem (Mumbai, India). All chemistry was performed under a nitrogen atmosphere using standard techniques. The chromatographic separations were performed over silica gel (230 to 400 mesh) using mixtures of EtOAc and methanol or EtOAc and hexane as eluent. Solvents were removed under reduced pressure on a rotovap. Organic extracts were dried with anhydrous Na_2_SO_4_. Visualization of spots on TLC plates was effected by UV illumination, exposure to iodine vapor and heating the plates dipped in KMnO_4_ stain.

### General procedure to synthesize pyrazolones from ketones

LiHMDS (1.0 M solution in toluene, 11 mmol) was added quickly to a solution of ketone (10 mmol in toluene (15 mL) using a syringe at 0°C under stirring and stirred at this temperature for 10 min; then, ethyl chloroformate (11 mmol) was added quickly. Reaction mixture was slowly (10 min) brought to room temperature and stirred for 10 min; then, 2 mL of acetic acid, 15 mL of ethanol and hydrazine hydrate (30 mmol) were added and refluxed for 15 min. Reaction mixture was concentrated to dryness under reduced pressure and re-dissolved in ethyl acetate, the organic layer was washed with saturated brine solution, dried over Na_2_SO_4_ and evaporated under reduced pressure. Crude product was purified by recrystallisation using ethanol.

#### 3-(4-Methoxyphenyl)-1H-pyrazol-5(4H)-one (1)

Purified by recrystallisation using ethanol (white solid), m.p: 221.0°C to 222.3°C, ^1^H NMR (400 MHz, *d*_6_-DMSO) *δ*_H_: 3.76 (s, 3H, methyl protons of -OCH_3_), 5.77 (s, 1H, proton at C-4), 6.95 (d, *J* = 8.80 Hz, 2 Hz, 2H, aryl protons), 7.57 (dd, *J* = 6.88 Hz and 1.92 Hz, 2H, aryl protons), 9.70 (bs, 1H, -NH proton), 11.90 (bs, 1H, -OH proton); ^13^C NMR (100 MHz, *d*_6_-DMSO): *δ* 55.19 (carbon at -OCH_3_), 86.26 (C-3), 114.20, 123.15, 126.17, 143.09 (aryl carbons), 158.94 (C-4), 161.21 (C-5). MS calculated for C_10_H_10_N_2_O_2_: 190.19. Found: 189.0 (M-1).

#### 3-(4-Chlorophenyl)-1H-pyrazol-5(4H)-one (2)

Purified by recrystallisation using ethanol (white solid), m.p: 243.5°C to 245.0°C, ^1^H NMR (400 MHz, *d*_6_-DMSO) *δ*_H_: 5.93 (s, 1H, proton at C-4), 7.46 (d, *J* = 6.80 Hz, 2H, aryl protons), 7.69 (d, *J* = 8.40 Hz, 2H, aryl protons), 9.70 (bs, 1H, -NH proton), 12.15 (bs, 1H, -OH proton); ^13^C NMR (100 MHz, *d*_6_-DMSO): *δ* 86.82 (C-4), 126.44, 128.78, 132.10 (aryl carbons), 142.0 (C-3), 160.70 (C-5). MS calculated for C_9_H_7_ClN_2_O: 194.61. Found: 195.0 (M + 1 for Cl^35^) and 197.0 (M + 3 for Cl^37^).

#### 3-(4-Fluorophenyl)-1H-pyrazol-5-(4H)-one (3)

Purified by recrystallisation using ethanol (white solid), m.p: 240.0°C to 241.5°C, ^1^H NMR (400 MHz, *d*_6_-DMSO) *δ*_H_: 5.86 (s, 1H, proton at C-4), 7.23 (t, *J* = 8.72 Hz, 2H, aryl protons), 7.69 (dd, *J* = 8.30 and 7.23 Hz, 2H, aryl protons), 9.70 (bs, 1H, -NH proton), 12.00 (bs, 1H, -OH proton); ^13^C NMR (100 MHz, *d*_6_-DMSO): *δ* 86.71 (C-4), 115.52, 115.74, 126.82, 126.74 (aryl carbons), 160.41 (C-4), 162.84 (C-5). MS calculated for C_9_H_7_FN_2_O: 178.10. Found: 177.0 (M-1).

#### 4-Methyl-3-phenyl-1H-pyrazol-5(4H)-one (4)

Purified by recrystallisation using ethanol (white solid), m.p: 218.5°C to 220.0°C, ^1^H NMR (400 MHz, *d*_6_-DMSO) *δ*_H_: 1.99 (s, 3H, methyl protons at C-4), 7.34 (t, *J* = 7.20 Hz, 1H, *para* proton of aryl), 7.45 (t, *J* = 8.00 Hz, 2H, *meta* protons of aryl), 7.53 (d, *J* = 8.00 Hz, 2H, *ortho* protons of aryl), 9.50 (bs, 1H, -NH proton), 11.70 (bs, 1H, -OH proton); ^13^C NMR (100 MHz, *d*_6_-DMSO): *δ* 7.66 (methyl carbon at C-4), 95.98 (C-4), 126.31, 127.49, 128.78, 131.15 (aryl carbons), 139.54 (C-3), 160.28 (C-5). MS calculated for C_10_H_10_N_2_O: 174.19. Found: 173.0 (M-1).

#### 4,5,6,7-Tetrahydro-2H-indazol-3(3aH)-one (5)

Purified by recrystallisation using ethanol (white solid), m.p: 286.0°C to 288.0°C, ^1^H NMR (400 MHz, *d*_6_-DMSO) *δ*_H_: 1.66 to 1.59 (m, 4H, four protons of cyclohexane fused ring), 2.21 (t, *J* = 5.20 Hz, 2H, two protons of cyclohexane fused ring), 2.42 (t, *J* = 6.0 Hz, 2H, two protons of cyclohexane fused ring), 9.95 (bs, 2H); ^13^C NMR (100 MHz, *d*_6_-DMSO): *δ* 19.35, 21.73, 22.74, 23.32 (carbons of fused cyclohexane part), 98.88 (C-3 of pyrazole ring), 140.19 (C-4 of pyrazole ring), 158.87 (C-5 of pyrazole ring). MS calculated for C_7_H_10_N_2_O: 138.08. Found: 138.16 (M+).

#### 3a,4,5,6,7,8-Hexahydrocyclohepta(e)pyrazol-3-(2H)-one (6)

Purified by recrystallisation using ethanol (white solid), m.p: 220.5°C to 221.8°C, ^1^H NMR (400 MHz, *d*_6_-DMSO) *δ*_H_: 1.56 to 1.50 (m, 4H, four protons of fused cycloheptane), 1.71 (d, *J* = 5.52 Hz, 2H, two protons of fused cycloheptane), 2.29 (t, *J* = 5.60 Hz, 2H, two protons of fused cycloheptane), 2.50 (t, *J* = 3.28 Hz, 2H, two protons of fused cycloheptane), 9.20 (bs, 1H, -NH proton), 11.00 (bs, 1H, -OH proton); ^13^C NMR (100 MHz, *d*_6_-DMSO): *δ* 23.04, 27.68, 29.36, 32.01 (carbons of fused cycloheptane ring), 102.81 (C-4) 143.81 (C-3 of pyrazole ring), 159.25 (C-5). MS calculated for C_8_H_12_N_2_O: 152.19. Found: 153.0 (M + 1).

#### 4,5,6,7,8,9-Hexahydro-2H-cycloocta(c)pyraol-3(3aH)-one (7)

Purified by recrystallisation using ethanol (white solid), m.p: 221.6°C to 228.8°C, ^1^H NMR (400 MHz, *d*_6_-DMSO) *δ*_H_: 1.40 (m, 4H, protons of fused cyclooctane ring), 1.51 (m, 2H, protons of fused cyclooctane ring), 1.58 (m, 2H, protons of fused cyclooctane ring), 2.34 (t, *J* = 6.2 Hz, 2H, protons of fused cyclooctane ring), 2.54 (t, *J* = 6.2 Hz, 2H, protons of fused cyclooctane ring), 9.03 (bs, 1H, -NH proton), 11.00 (bs, 1H, -OH proton); ^13^C NMR (100 MHz, *d*_6_-DMSO): *δ* 20.13, 24.27, 25.50, 25.76, 28.76, 28.94 (carbons of fused cyclooctane ring), 100.42 (C-4), 141.62 (C-3), 159.42 (C-5). MS calculated for C_9_H_14_N_2_O: 166.20. Found: 167.0 (M + 1).

#### 3-Cyclohexyl-1H-pyrazol-5(4H)-one (8)

Purified by recrystallisation using ethanol (white solid), m.p: 241.5°C to 243.0°C, ^1^H NMR (400 MHz, *d*_6_-DMSO) *δ*_H_: 1.21 to 1.26 (m, 1H, proton of cyclohexyl ring), 1.29 to 1.34 (m, 4H, protons of cyclohexyl ring), 1.63 to 1.71 (m, 3H, protons of cyclohexyl ring), 1.84 to 1.90 (m, 2H, protons of cyclohexyl ring)), 2.44 to 2.50 (m, 1H, proton at C1′ of cyclohexyl ring), 5.20 (s, 1H, proton at C-4), 9.30 (bs, 1H, -NH proton), 11.00 (bs, 1H, -OH proton); ^13^C NMR (100 MHz, *d*_6_-DMSO) *δ*: 25.97, 26.08, 32.66, 35.60 (carbons of cyclohexyl ring), 86.74 (C-4), 149.83 (C-3), 161.17 (C-5). MS calculated for C_9_H_14_N_2_O: 166.22. Found: 166.9 (M+).

#### 3-(3-Chloropropyl)-1H-pyrazol-5(4H)-one (9)

Purified by recrystallisation using ethanol (white solid), m.p: 155.8°C to 156.5°C, ^1^H NMR (400 MHz, *d*_6_-DMSO) *δ*_H_: 2.00 to 1.93 (m, 2H, methylene protons at C2′ of propyl), 2.57 (t, *J* = 7.36 Hz, 2H, methylene protons at C1′ of propyl), 3.62 (t, 6.40 Hz, 2H, methylene protons at C3′ of propyl), 5.25 (s, 1H), 9.50 (bs, 1H, -NH proton), 11.20 (bs, 1H, -OH proton); ^13^C NMR (100 MHz, *d*_6_-DMSO): *δ* 23.51 (C2′ of propyl), 37.18 (C1′ of propyl), 45.13 (C3′ of propyl), 88.56 (C-4), 143.39 (C-3), 161.20 (C-5). MS calculated for C_6_H_9_ClN_2_O: 160.60. Found: 161.0 (M + 1 for Cl^35^) and 163.60 (M + 3 for Cl^37^).

#### 7-Methoxy-4,5-dihydro-2H-benzo(g)indazol-23(3aH)-one (10)

Purified by recrystallisation using ethanol (white solid), m.p: 116.4°C to 118.2°C, ^1^H NMR (400 MHz, *d*_6_-DMSO) *δ*_H_: 2.50 (t, *J* = 5.50 Hz, 2H, protons of cyclohexyl B ring), 2.82 (t, *J* = 5.6 Hz, 2H, protons of cyclohexyl B ring), 3.75 (s, 3H, protons of methoxy group), 6.80 (d, *J* = 7.0 Hz, 1H, aryl proton of C ring), 6.86 (s, 1H, aryl proton of C ring), 7.43 (d, *J* = 7.0 Hz, 1H, aryl proton of C ring), 9.50 (bs, 1H, -NH); ^13^C NMR (100 MHz, *d*_6_-DMSO): *δ* 17.91, 30.04, 30.52 (carbons of cyclohexyl B ring), 55.52 (methoxy carbon), 97.83 (C-4), 112.20, 114.59, 114.81, 120.61, 122.61, 138.18, 139.82, 157.82 (C-3), 158.97 (C-5). MS calculated for C_12_H_12_N_2_O_2_: 216.23. Found: 215.0 (M-1).

#### 3-(2,3-Dihydrobenzofuran-5-yl)-1H-pyrazol-5(4H)-one (11)

Purified by recrystallisation using ethanol (white solid), m.p: 237.5°C to 239.0°C, ^1^H NMR (400 MHz, *d*_6_-DMSO) *δ*_H_: 3.19 (t, *J* = 8.70 Hz, 2H, protons of benzofuran ring), 4.54 (t, *J* = 8.70 Hz, 2H, protons of benzofuran ring), 5.74 (s, 1H, proton at C-4), 6.67 (d, *J* = 8.28 Hz, 1H, aryl proton of benzofuran ring), 7.38 (dd, *J* = 8.28 Hz, 1.82 Hz, 1H, aryl proton of benzofuran ring), 7.51 (s, 1H, aryl proton of benzofuran ring), 9.65 (bs, 1H, -NH proton), 11.85 (bs, 1H, -OH proton); ^13^C NMR (100 MHz, *d*_6_-DMSO): *δ* 28.95 (C of benzofuran), 71.14 (C of benzofuran), 86.16 (C-3), 109.05, 121.84, 123.09, 124.77, 127.95 (carbons of benzofuran), 143.51 (C-3), 159.50 (carbon of benzofuran), 161.16 (C-5). MS calculated for C_11_H_10_N_2_O_2_: 202.20. Found: 203.0 (M + 1).

#### 3-(Biphenyl-4-yl)-1H-pyrazol-5(4H)-one (12)

Purified by recrystallisation using ethanol (white solid), m.p: 236.5°C to 265.0°C, ^1^H NMR (400 MHz, *d*_6_-DMSO) *δ*_H_: 5.94 (s, 1H, proton at C-4), 7.37 (t, *J* = 7.5 Hz, 1H, aryl proton), 7.47 (t, *J* = 7.5 Hz, 2H, aryl protons), 7.76 to 7.69 (m, 6H, aryl protons), 9.77 (bs, 1H, -NH proton), 12.13 (bs, 1H, -OH proton); ^13^C NMR (100 MHz, *d*_6_-DMSO): *δ* 86.88 (C-4), 125.26, 126.51, 126.97, 127.54, 128.96 (aryl carbons), 139.25 (C-3), 139.50 (C-5). MS calculated for C_15_H_10_N_2_O_2_: 236.26. Found: 235.0 (M-1).

#### 3-(Thiophen-2-yl)-1H-pyrazol-5(4H)-one (13)

Purified by recrystallisation using ethanol (white solid), m.p: 204.0°C to 205.0°C, ^1^H NMR (400 MHz, *d*_6_-DMSO) *δ*_H_: 5.67 (s, 1H, proton at C-4), 7.07 (bs, 1H, proton of thiophenyl ring), 7.32 (bs, 1H, proton of thiophenyl ring), 7.42 (bs, 1H, proton of thiophenyl ring), 9.67 (bs, 1H, -NH), 12.05 (bs, 1H, -OH). MS calculated for C_7_H_6_N_2_OS: 166.20. Found: 167.0 (M + 1).

#### 3-(5-Oxo-4,5-dihydro-1H-pyrazol-3-yl)benzonitrile (14)

Purified by recrystallisation using ethanol (white solid). ^1^H NMR (400 MHz, *d*_6_-DMSO) *δ*_H_: 6.02 (s, 1H, proton at C-4), 7.59 (t, *J* = 10.4 Hz, 1H, aryl proton), 7.73 (d, *J* = 10.4 Hz, 1H, aryl proton), 7.99 (d, *J* = 10.4, 1H, aryl proton), 8.12 (s, 1H, aryl proton), 10.00 (bs, 1H, -NH proton), 12.02 (bs, 1H, -OH proton). MS calculated for C_10_H_7_N_3_O: 185.18. Found: 184.0 (M-1).

#### Ethyl 3-oxo-2,3,3a,4,6,7-hexahydropyrazolo[4,3-c]pyridine-5-caboxylate (15)

Purified by recrystallisation using ethanol (white solid), m.p: 212.5°C to 213. 8°C, ^1^H NMR (400 MHz, *d*_6_-DMSO) *δ*_H_: 1.90 (t, *J* = 7.08 Hz, 3H, methyl of ethyl group), 2.50 (m, 2H, protons of ring B), 3.56 (t, *J* = 5.7 Hz, 2H, protons of ring B), 4.04 (q, *J* = 7.08 Hz, 2H, methylene of ethyl group), 4.18 (s, 2H, protons of ring B), 9.80 (bs, 1H, -NH proton), 11.30 (bs, 1H, -OH proton); ^13^C NMR (100 MHz, *d*_6_-DMSO): *δ* 14.62 (methyl carbon of ethyl group), 21.62 (carbon of ring B), 21.92 (methylene carbon of ethyl group), 60.89, 96.06 (C-4 of pyrazole ring), 138.12, 155.08 (C-3 of pyrazole ring), 156.33 (C-5 of pyrazole ring). MS calculated for C_9_H_13_N_3_O_3_: 211.21. Found: 212.0 (M + 1).

#### Tert-butyl 3-oxo-2,3,3a,4,6,7-hexahydropyrazolo[4,3-c]pyridine-5-carboxylate (16)

Purified by recrystallisation using ethanol (white solid), m.p: 225.5°C to 227.5°C, ^1^H NMR (400 MHz, *d*_6_-DMSO) *δ*_H_: 1.40 (s, 9H, methyl protons of Boc), 2.49 (t, *q* = 1.77 Hz, 2H, protons of ring B), 3.51 (t, *J* = 5.72 Hz, 2H, protons of ring B), 4.13 (s, 2H, protons of ring B); ^13^C NMR (100 MHz, *d*_6_-DMSO): *δ* 21.75 (carbons of B ring), (28.08 methyl carbons of Boc group), 59.77 (carbon of B ring), 78.94 (quaternary carbon of Boc), 96.21 (C-4 of pyrazole ring), 138.24 (C-3 carbon of pyrazole ring), 154.19 (C-5 carbon of pyrazole ring), 156.37 (carbonyl carbon of Boc). MS calculated for C_11_H_17_N_3_O_3_: 239.27. Found: 239.8 (M+).

#### 3-(2,5-Dimethylfuran-3-yl)-1H-pyrazol-5(4H)-one (17)

Purified by recrystallisation using ethanol (white solid), ^1^H NMR (400 MHz, *d*_6_-DMSO) *δ*_H_: 2.21 (s, 3H, methyl proton of furan ring), 2.32 (s, 3H, methyl proton of furan ring), 5.51 (s, 1H, proton at C-4) 6.27 (s, 1H, proton of furan ring), 9.60 (bs, 1H, -NH proton), 11.62 (bs, 1H, -OH proton). MS calculated for C_9_H_10_N_2_O_2_: 178.18. Found: 179. 0 (M + 1).

#### Benzyl 3-oxo-2,3,3a,4,6,7-hexahydropyrazolo[4,3-c]pyridine-5-carboxylate (18)

Purified by recrystallisation using ethanol (white solid), m.p: 225.4°C to 226.1°C, ^1^H NMR (400 MHz, *d*_6_-DMSO) *δ*_H_: 2.50 to 2.56 (m, 2H, protons of ring B), 3.61 (s, 2H, protons of ring B), 4.23 (d, *J* = 10.80 Hz, 2H, protons of ring B), 5.10 (s, 2H, protons of methylene of Cbz group), 7.38 to 7.30 (m, 5H, aryl protons of Cbz), 9.88 (bs, 1H, -NH proton), 11.16 (bs, 1H, -OH proton); ^13^C NMR (100 MHz, *d*_6_-DMSO): *δ* 41.27, 42.77, 66.39 (carbons of ring B), 86.72 (C-4), 127.69, 127.01, 128.46 (aryl carbons), 136.90 (C-3), 157.72 (C-5). MS calculated for C_14_H_15_N_3_O_3_: 273.28. Found: 273.8 (M+).

#### 5-Tert-butyl-4,5,6,7-tetrahydro-2H-indazol-3(3aH)-one (19)

Purified by recrystallisation using ethanol (white solid), m.p: 243.5°C to 244.8°C, ^1^H NMR (400 MHz, *d*_6_-DMSO) *δ*_H_: 0.89 (s, 9H, protons of three methyl groups), 1.18 to 1.25 (m, 2H, protons of ring B), 1.85 to 1.92 (m, 2H, protons of ring B), 2.39 to 2.29 (m, 2H, protons of ring B), 2.55 (m, 1H, proton of ring B); ^13^C NMR (100 MHz, *d*_6_-DMSO): *δ* 20.73 (carbons of methyl groups of tertiary group), 22.54, 24.48, 27.81, 27.85, 32.70, 45.50 (quaternary carbon of tertiary group), 99.42 (C-4 of pyrazole ring), 140.46 (C-3 of pyrazole ring), 158.88 (C-5 of pyrazole ring). MS calculated for C_11_H_18_N_2_O: 194.21. Found: 194.8 (M+).

#### 3-(Biphenyl-4-yl)-1-(4-fluorophenyl)-1H-pyrazol-5(4H)-one (20)

Purified by recrystallisation using ethanol (white solid), m.p: 156.2°C to 157.5°C, ^1^H NMR (400 MHz, *d*_6_-DMSO) *δ*_H_: 6.07 (s, 1H at C-4), 7.31 to 7.40 (m, 3H, aryl protons), 7.48 (t, *J* = 8.0 Hz, 2H, aryl protons), 7.07 to 7.33 (m, 4H, aryl protons), 7.83 to 7.93 (m, 4H, aryl protons), 11.94 (bs, 1H, -OH proton at C-5); ^13^C NMR (100 MHz, *d*_6_-DMSO): *δ* 85.57 (C-4), 116.01, 116.24, 123.52, 123.60, 126.10, 126.96, 127.25, 127.94, 129.42, 132.92, 135.74, 139.91, 140.17 (aryl carbons), 149.69 (C-3), 154.18, 159.05 (aryl carbons), 161.46 (C-5). MS calculated for C_21_H_15_FN_2_O: 330.55. Found: 329.0 (M-1).

#### 3-Ethyl-4-methyl-1H-pyrazol-5(4H)-one (21)

Purified by recrystallisation using ethanol (white solid), m.p: 233.4°C to 234.1°C, ^1^H NMR (400 MHz, *d*_6_-DMSO) *δ*_H_: 1.07 (t, *J* = 7.64 Hz, 3H, methyl protons of ethyl group), 1.72 (s, 3H, methyl at C-4), 2.40 (q, *J* = 7.6 Hz, 2H, methylene protons of ethyl group), 9.50 (bs, 1H, -OH proton), 10.05 (bs, 1H, -OH proton); ^13^C NMR (100 MHz, *d*_6_-DMSO): *δ* 11.34 (methyl carbon of ethyl group), 18.35 (methyl group at C-4), 22.99 (methylene carbon of ethyl group), 99.73 (C-4), 147.27 (C-3), 164.86 (C-5). MS calculated for C_6_H_10_N_2_O: 126.15. Found: 128.0 (M + 2).

#### 4-Ethyl-3-phenyl-1H-pyrazol-5(4H)-one (23)

Purified by recrystallisation using ethanol (white solid), m.p: 88.3°C to 89.1°C. ^1^H NMR (400 MHz, *d*_6_-DMSO) *δ*_H_: 1.15 (t, *J* = 7.6 Hz, 3H, protons of methyl group), 2.64 (q, *J* = 7.6 Hz, 2H, protons of methylene group), 7.17 to 7.13 (m, 1H), 7.41 to 7.32 (m, 3H), 10.00 (bs, 2H, -OH and -NH protons); ^13^C NMR (100 MHz, *d*_6_-DMSO): *δ* 13.57 (methyl carbon of ethyl group), 19.10 (methylene carbon of ethyl group), 102.27 (C-4), 125.34 (*ipso*), 128.12 (*ortho*), 128.60 (*meta*), 133.88 (*para*), 142.49 (C-3), 159.20 (C-5). MS calculated for C_11_H_12_N_2_O: 188.22. Found: 188.8 (M+).

#### 3-Cyclohexyl-4-methyl-1H-pyrazol-5(4H)-one (24)

Purified by recrystallisation using ethanol (white solid), m.p: 205.4°C to 206.2°C. ^1^H NMR (400 MHz, *d*_6_-DMSO) *δ*_H_: 1.25 to 1.28 (m, 1H, proton of cyclohexyl ring) 1.32 to 1.40 (m, 4H, protons of cyclohexyl ring), 1.66 to 1.76 (m, 8H, 5 protons of cyclohexyl ring and protons of methyl group), 2.40 to 2.50 (m, 1H, proton of cyclohexyl ring), 9.50 (bs, 1H, -NH proton) 10.52 (bs, 1H, -OH proton); ^13^C NMR (100 MHz, *d*_6_-DMSO): *δ* 6.91 (carbon of methyl group), 26.01, 26.53, 31.91, 36.42 (carbons of cyclohexyl ring), 94.38 (C-4), 145.71 (C-3), 160.12 (C-5). MS calculated for C_10_H_16_N_2_O_2_: 180.24. Found: 180.8 (M+).

#### 3-Cyclopropyl-1H-pyrazol-5(4H)-one (25)

Purified by recrystallisation using ethanol, m.p: 215.5°C to 216.8°C (white solid).^1^H NMR (400 MHz, *d*_6_-DMSO) *δ*_H_: 0.58 to 0.55 (m, 2H, protons of cyclopropyl), 0.85 to 0.81 (m, 2H, protons of cyclopropyl), 1.75 to 1.68 (m, 1H, proton of cyclopropyl), 9.50 (bs, 1H, -NH proton), 11.52 (bs, 1H, -OH proton); ^13^C NMR (100 MHz, *d*_6_-DMSO): *δ* 7.27 (C-1′ of cyclopropyl ring), 7.59 (C-2′, 3′ of cyclopropyl ring), 85.78 (C-4), 146.75 (C-3), 160.78 (C-5). MS calculated for C_6_H_8_N_2_O: 124.14. Found: 124.9 (M+).

#### 3-Isopropyl-1H-pyrazol-5(4H)-one (26)

Purified by recrystallisation using ethanol, m.p: 198.2°C to 199.4°C (white solid).^1^H NMR (400 MHz, *d*_6_-DMSO) *δ*_H_: 1.13 (d, *J* = 6.92 Hz, 6H), 2.79 to 2.72 (m, 1H), 5.20 (s, 1H), 9.32 (bs, 1H, -NH proton), 11.50 (bs, 1H, -OH proton); ^13^C NMR (100 MHz, *d*_6_-DMSO): *δ* 22.24 (carbon of two CH_3_ of *iso*-propyl), 25.69 (methine carbon of *iso*-propyl), 86.22 (C-4), 150.39 (C-3), 160.75 (C-5). MS calculated for C_6_H_10_N_2_O: 126.15. Found: 126.9 (M+).

## Conclusions

The β-keto esters from ethyl chloroformate was successfully attempted, and the developed method is simple, fast and applicable to the ketones having the alkyl halogens, protecting groups like Boc and Cbz that were tolerated and proved to be useful in the synthesis of fused bicyclic and tricyclic pyrazolones efficiently using cyclic ketones. Since this method is successful for different ketones, it can be useful for the synthesis of pharmaceutically important pyrazolones also. All the new pyrazolones were subjected to antimicrobial, docking and cytotoxicity assay against ACHN (human renal cell carcinoma), Panc-1 (human pancreatic adenocarcinoma) and HCT-116 (human colon cancer) cell line. Most of them were found to be active against different bacterial and fungal strains tested, and some of them were found to have promising activity. The *in silico* and cytotoxicity studies reveal that compound **18** was found to be inhibitive against only ACHN (human renal cell carcinoma) cell lines. The compounds **1** and **10** were found to be inhibitive against HCT-116 (human colon cancer) cell lines. The compound **14** was found to be inhibitive against Panc-1 (human pancreatic adenocarcinoma) as well as HCT-116 (human colon cancer) cell lines, and hence, further investigations are in need in these promising lead molecules.

## Competing interests

The authors declare that they have no competing interests.

## Supplementary Material

Additional file 1 **Spectral evidences.** A copy of original ^1^H NMR and ^13^C NMR spectra of the compounds **1** to **26** has been included. Click here for file
