# Supplementary material for: β-Keto esters from ketones and ethyl chloroformate: a rapid, general, efficient synthesis of pyrazolones and their antimicrobial, in silico and in vitro cytotoxicity studies
Source: Org Med Chem Lett. 2013 Jul 19;3:6. doi: 10.1186/2191-2858-3-6 (PMC3726461; doi:10.1186/2191-2858-3-6)
Supplement: Additional file 1 — Spectral evidences. A copy of original 1H NMR and 13C NMR spectra of the compounds 1 to 26 has been included. [file 2191-2858-3-6-S1.doc]

SPECTRAL EVIDENCES


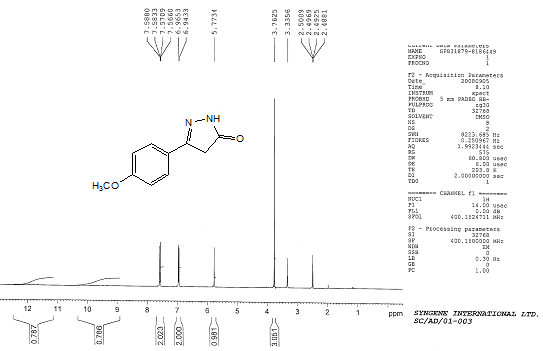


Fig.1 ^1^H NMR spectrum of 3-(4-Methoxyphenyl)-1*H*-pyrazol-5(4*H*)-one **(1)**


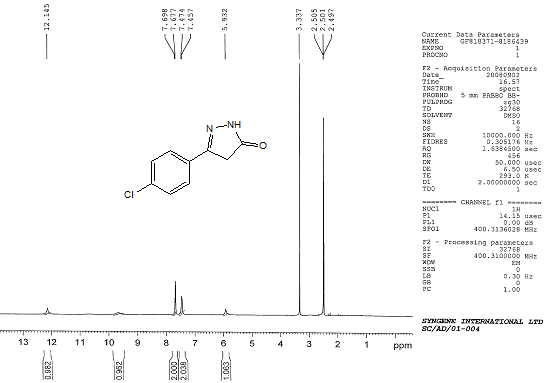


Fig.2 ^1^H NMR spectrum of 3-(4-Chlorophenyl)-1*H*-pyrazol-5(4*H*)one **(2)**


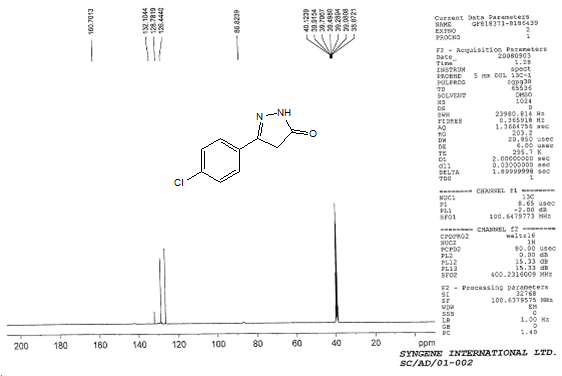


Fig.3 ^13^C NMR spectrum of 3-(4-Chlorophenyl)-1*H*-pyrazol-5(4*H*)one **(2)**


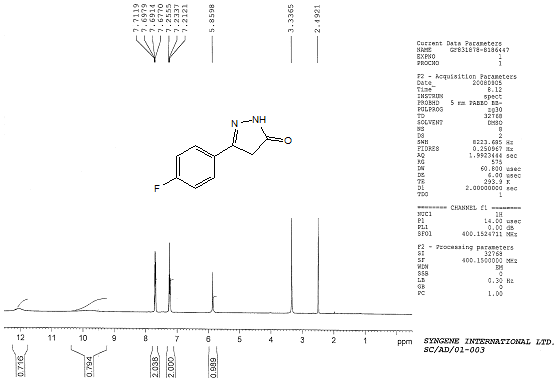


Fig.4 ^1^H NMR spectrum of 3-(4-Fluorophenyl)-1*H*-pyrazol-5-(4*H*)-one **(3)**


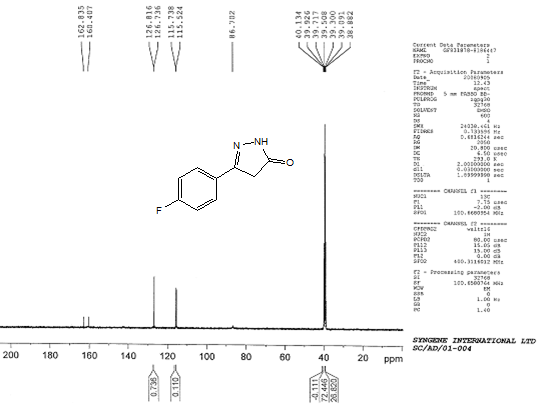


Fig.5 ^13^C NMR spectrum of 3-(4-Fluorophenyl)-1*H*-pyrazol-5-(4*H*)-one **(3)**


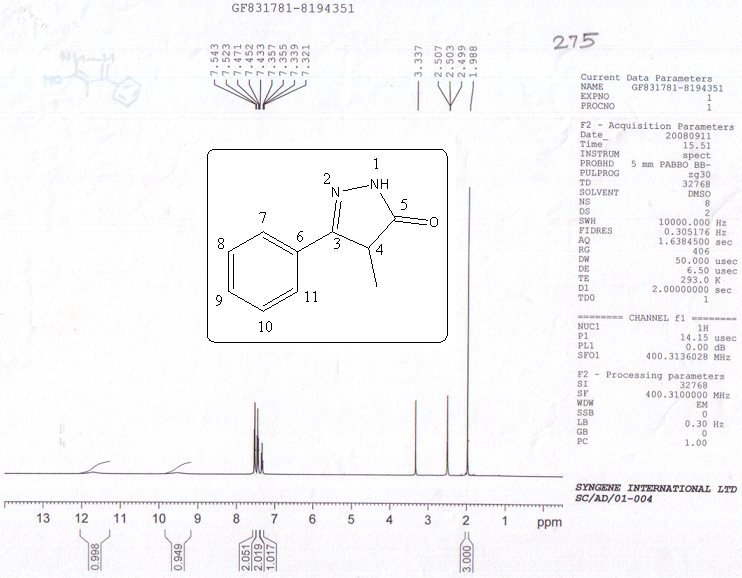


Fig.6 ^1^H NMR spectrum of 4-methyl-3-phenylpyrazol-5-(4*H*)-one **(4)**


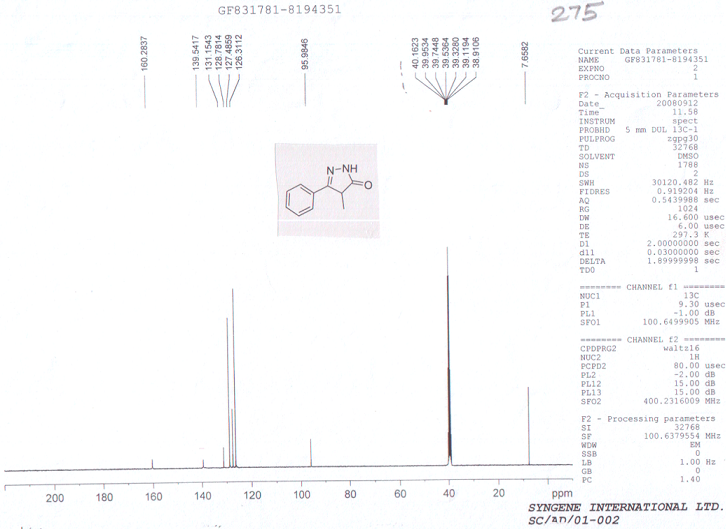


Fig.7 ^13^C NMR spectrum of 4-methyl-3-phenylpyrazol-5-(4*H*)-one **(4)**


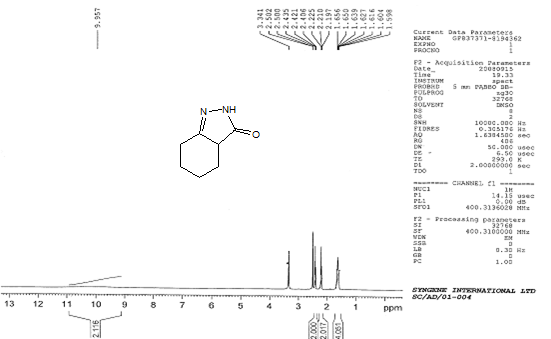


Fig.8 ^1^H NMR spectrum of 4,5,6,7-Tetrahydro-2*H*-indazol-3(3a*H*)-one **(5)**


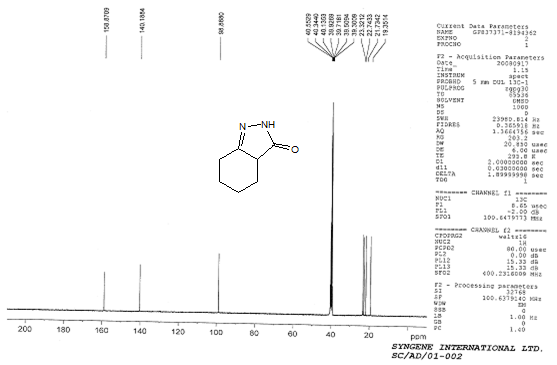


Fig.9 ^13^C NMR spectrum of 4,5,6,7-Tetrahydro-2*H*-indazol-3(3a*H*)-one **(5)**


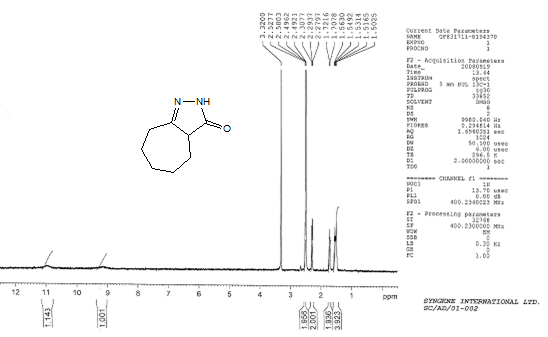


Fig.10 ^1^H NMR spectrum of 3a,4,5,6,7,8-Hexahydro-2*H*-cyclohepta[e]pyrazol-3(2*H*)-one **(6)**


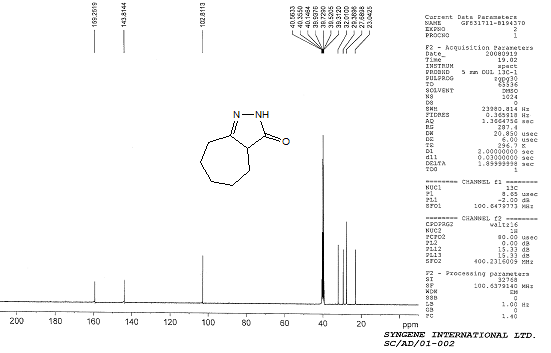


Fig.11. ^13^C NMR spectrum of 3a,4,5,6,7,8-Hexahydro-2*H*-cyclohepta[e]pyrazol-3(2*H*)-one **(6)**


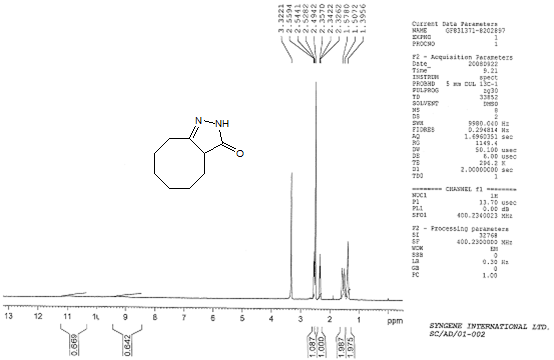


Fig.12. ^1^H NMR spectrum of 4,5,6,7,8,9-Hexahydro-2*H*-cycloocta[c]pyrazol-3(3a*H*)-one **(7)**


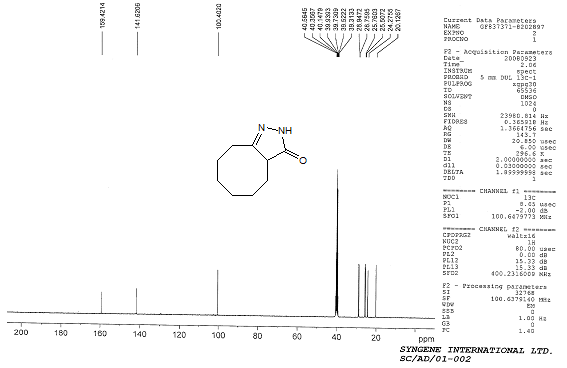


Fig.13. ^13^C NMR spectrum of 4,5,6,7,8,9-Hexahydro-2*H*-cycloocta[c]pyrazol-3(3a*H*)-one (**7**)


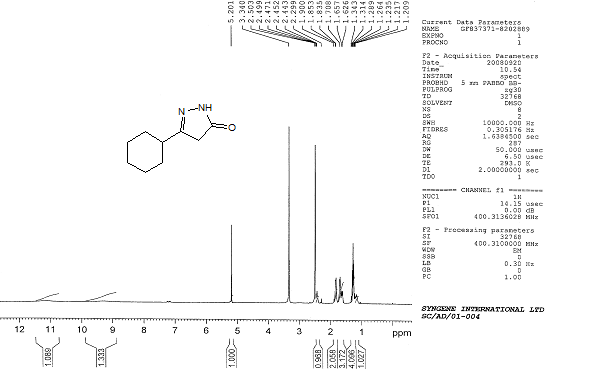


Fig.14 ^1^H NMR spectrum of 3-Cyclohexyl-1*H*-pyrazol-5(4*H*)-one **(8)**


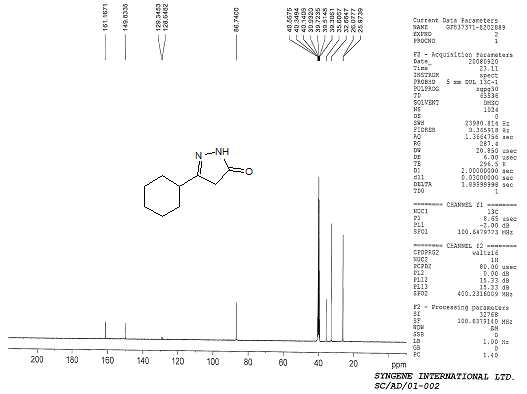


Fig.15. ^13^C NMR spectrum of 3-Cyclohexyl-1*H*-pyrazol-5(4*H*)-one **(8)**


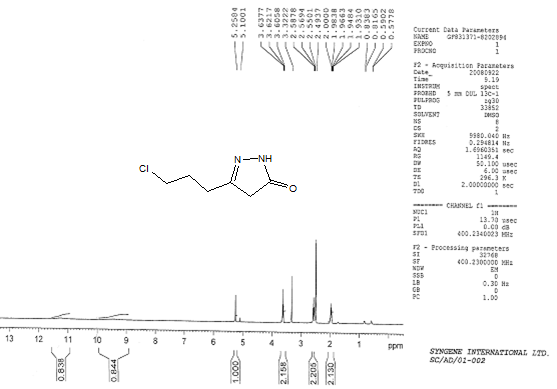


Fig.16. ^1^H NMR spectrum of 3-(3-Chloropropyl)-1*H*-pyrazol-5(4*H*)-one **(9)**


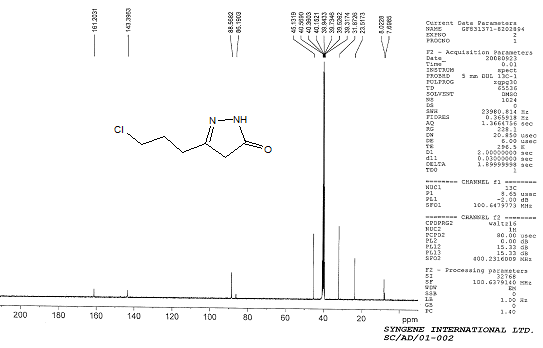


Fig.17. ^13^C NMR spectrum of 3-(3-Chloropropyl)-1*H*-pyrazol-5(4*H*)-one **(9)**


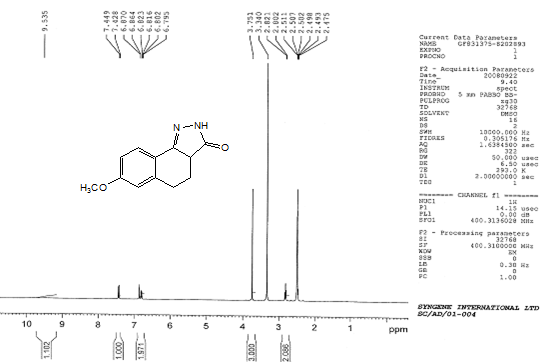


Fig.18. ^1^H NMR spectrum of 7-Methoxy-4,5-dihydro-2*H*-benzo[g]indazol-23(3a*H*)-one **(10)**


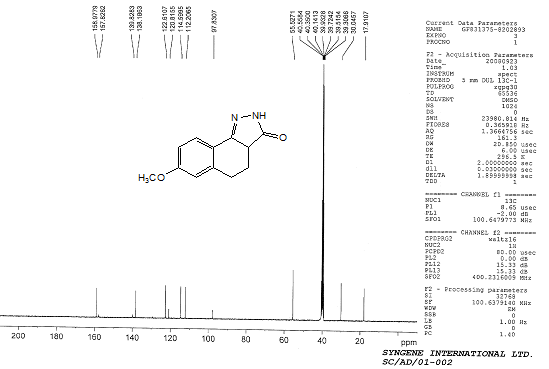


Fig.19. ^13^C NMR spectrum of 7-Methoxy-4,5-dihydro-2*H*-benzo[g]indazol-23 (3a*H*)-one **(10)**


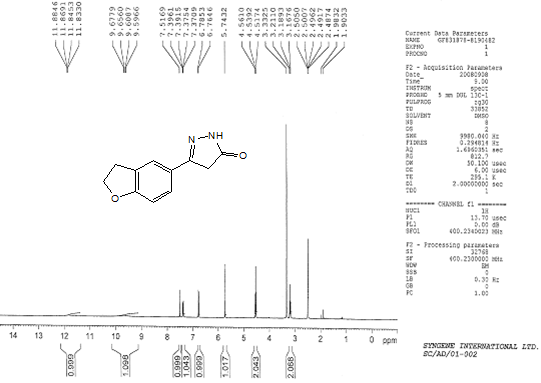


Fig.20. ^1^H NMR spectrum of 3-(2,3-Dihydrobenzofuran-5-yl)-1*H*-pyrazol-5(4*H*)-one **(11)**


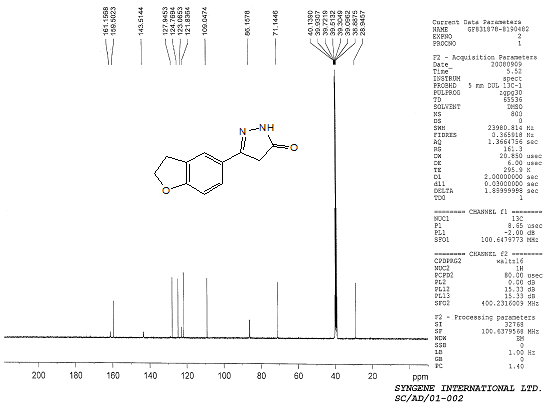


Fig.21. ^13^C NMR spectrum of 3-(2,3-Dihydrobenzofuran-5-yl)-1*H*-pyrazol-5(4*H*)-one **(11)**


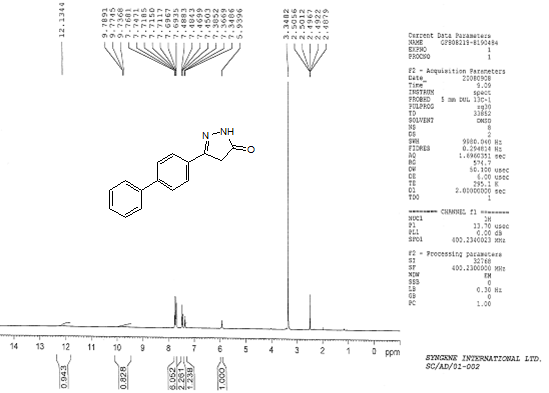


Fig.22. ^1^H NMR spectrum of 3-(Biphenyl-4-yl)-1*H*-pyrazol-5(4*H*)-one **(12)**


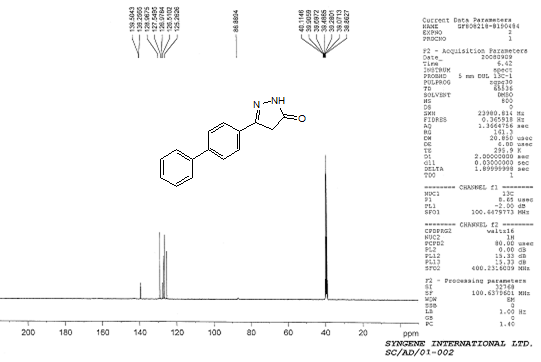


Fig.23. ^13^C NMR spectrum of 3-(Biphenyl-4-yl)-1*H*-pyrazol-5(4*H*)-one **(12)**

**
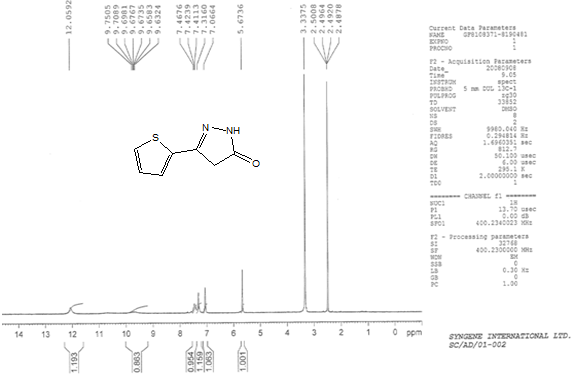
**

Fig.24 ^1^H NMR spectrum of 3-(Thiophen-2-yl)-1*H*-pyrazol-5(4*H*)-one **(13)**

**
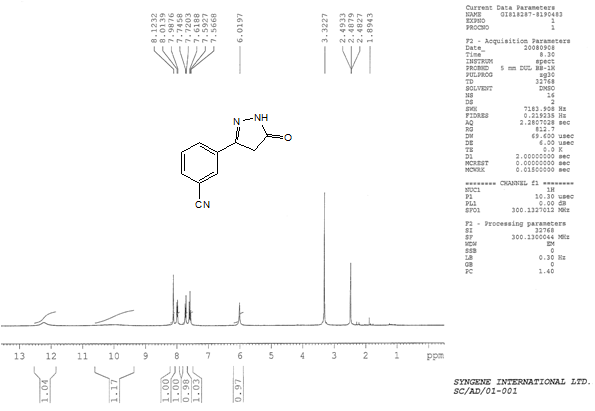
**

Fig.25. ^1^H NMR spectrum of 3-(5-Oxo-4,5-dihydro-1*H*-pyrazol-3-yl)benzonitrile **(14)**

**
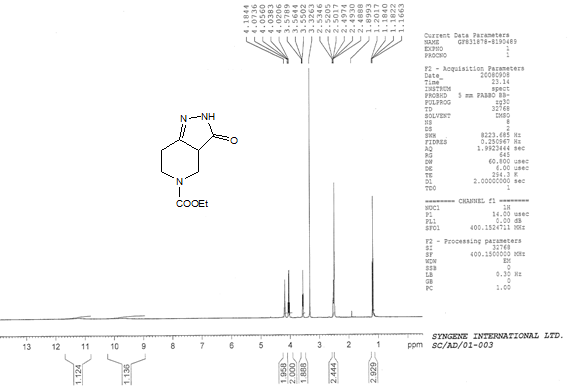
**

Fig.26. ^1^H NMR spectrum of Ethyl 3-oxo-2,3,3a,4,6,7-hexahydropyrazolo[4,3-c] pyridine-5-caboxylate **(15)**


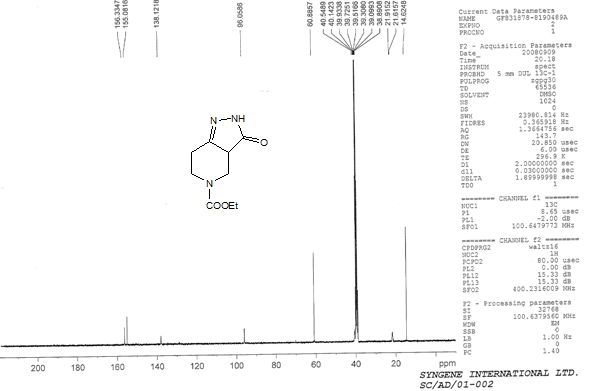


Fig.27. ^13^C NMR spectrum of Ethyl 3-oxo-2,3,3a,4,6,7-hexahydropyrazolo[4,3-c] pyridine-5-caboxylate **(15)**

**
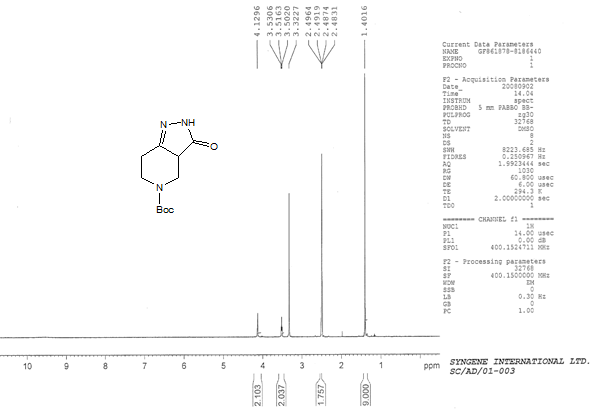
**

Fig.28. ^1^H NMR Spectrum of *Tert*-butyl 3-oxo-2,3,3a,4,6,7-hexahydropyrazolo[4,3-c]pyridine-5-carboxylate **(16).**

**
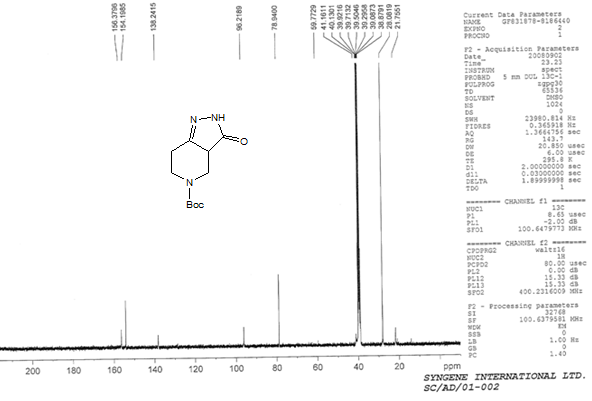
**

Fig.29. ^13^C NMR spectrum of *Tert-*butyl 3-oxo-2,3,3a,4,6,7-hexahydropyrazolo[4,3-c] pyridine-5-carboxylate **(16).**

**
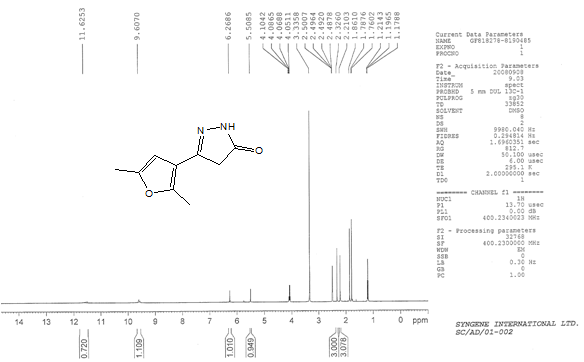
**

Fig.30. ^1^H NMR spectrum of 3-(2,5-Dimethylfuran-3-yl)-1*H*-pyrazol-5(4*H*)-one **(17).**

**
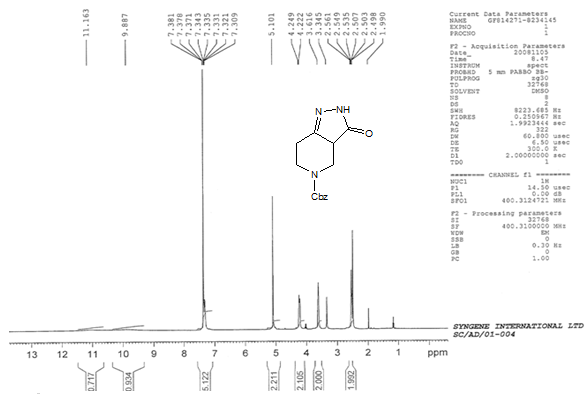
**

Fig.31. ^1^H NMR spectrum of Benzyl 3-oxo-2,3,3a,4,6,7-hexahydropyrazolo(4,3-c) pyridine-5-carboxylate **(18).**


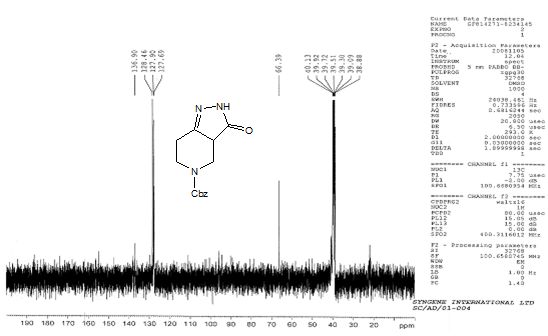


Fig.32. ^13^C NMR spectrum of Benzyl 3-oxo-2,3,3a,4,6,7-hexahydropyrazolo(4,3-c) pyridine-5-carboxylate **(18).**


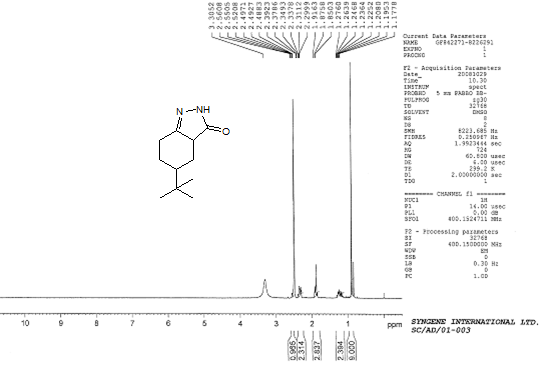


Fig.33. ^1^H NMR spectrum of 5-*Tert*-butyl-4,5,6,7-tetrahydro-2*H*-indazol-3(3a*H*)-one **(19)**


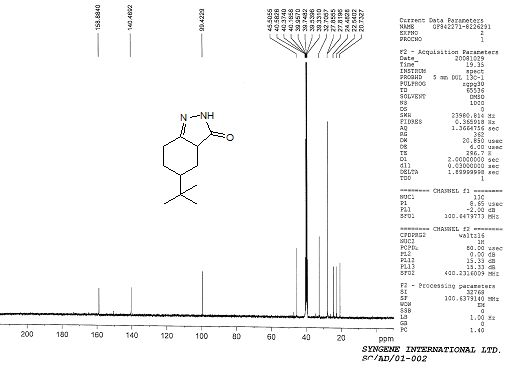


Fig.34. ^13^C NMR spectrum of 5-*Tert*-butyl-4,5,6,7-tetrahydro-2*H*-indazol-3(3a*H*)-one **(19)**


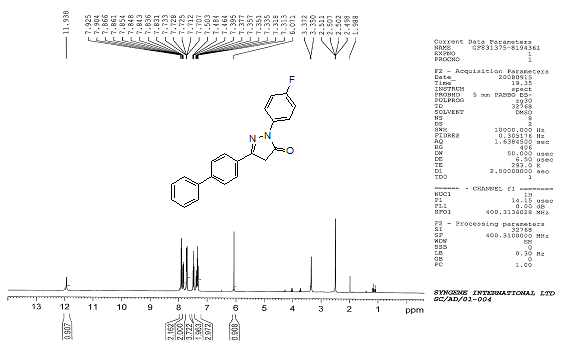


Fig.35. ^1^H NMR spectrum of 3-(Biphenyl-4-yl)-1-(4-fluorophenyl)-1*H*-pyrazol-5(4*H*)-one **(20)**


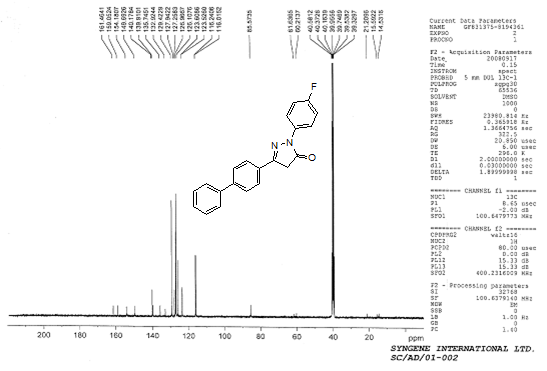


Fig.36. ^13^C NMR spectrum of 3-(Biphenyl-4-yl)-1-(4-fluorophenyl)-1*H*-pyrazol-5(4*H*)-one **(20).**


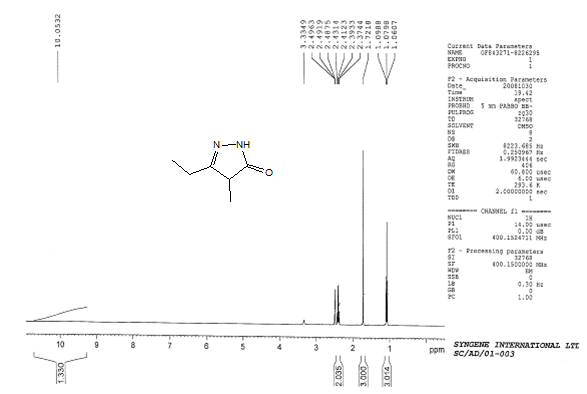


Fig.37. ^1^H NMR spectrum of 3-Ethyl-4-methyl-1*H*-pyrazol-5(4*H*)one **(21)**


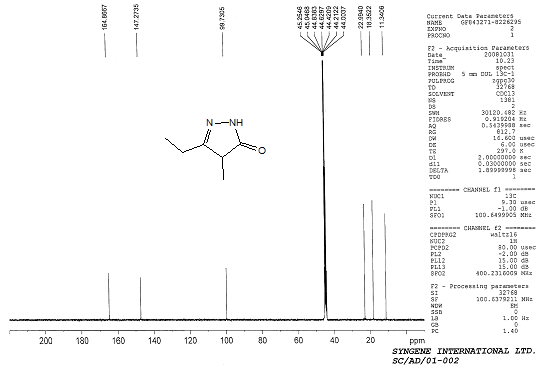


Fig.38. ^13^C NMR spectrum of 3-Ethyl-4-methyl-1*H*-pyrazol-5(4*H*)one **(21)**


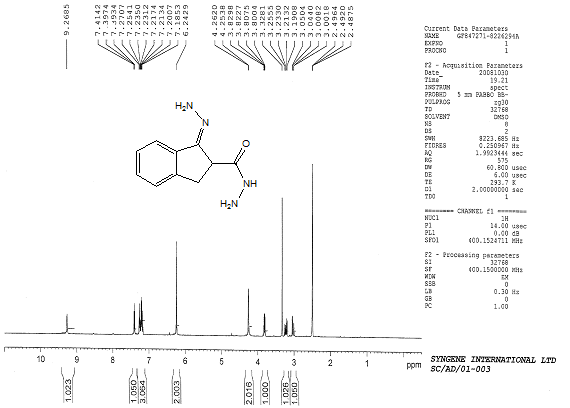


Fig.39. ^1^H NMR spectrum of (E)-1-Hydrazono-2,3-dihydro-1*H*-indole-2-carbohydrazide **(22)**

**
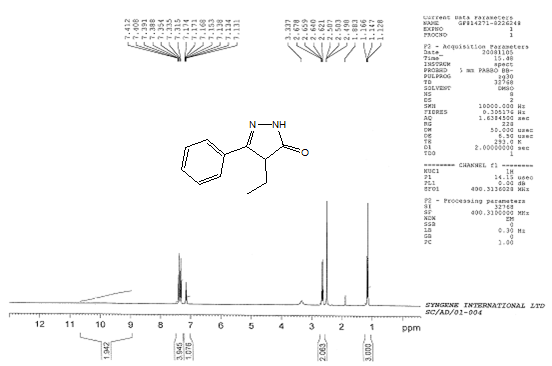
**

Fig.40. ^1^H NMR spectrum of 4-Ethyl-3-phenyl-1*H*-pyrazol-5(4*H*)-one **(23)**


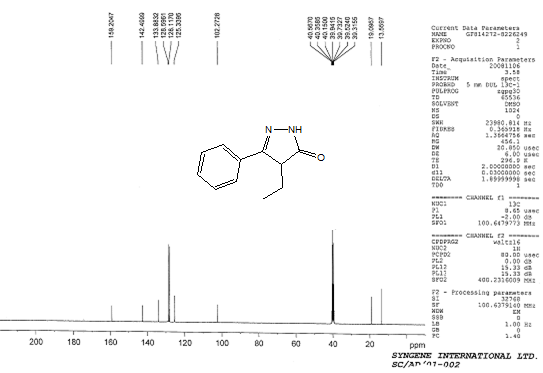


Fig.41. ^13^C NMR spectrum of 4-Ethyl-3-phenyl-1*H*-pyrazol-5(4*H*)-one **(23)**


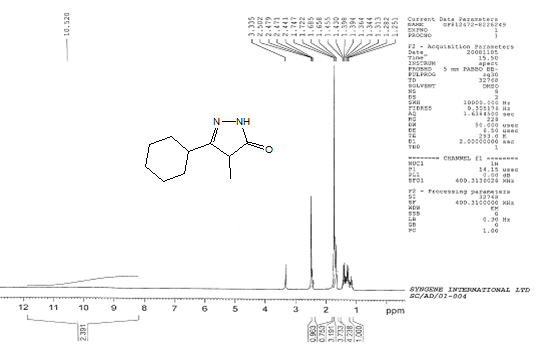


Fig.42. ^1^H NMR spectrum of 3-Cyclohexyl-4-methyl-1*H*-pyrazol-5(4*H*)-one **(24)**


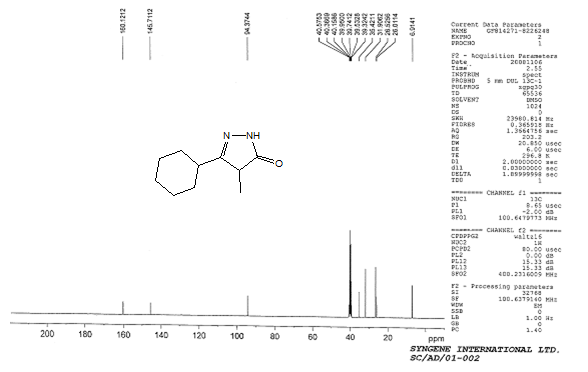


Fig.43. ^13^C NMR spectrum of 3-Cyclohexyl-4-methyl-1*H*-pyrazol-5(4*H*)-one **(24)**

**
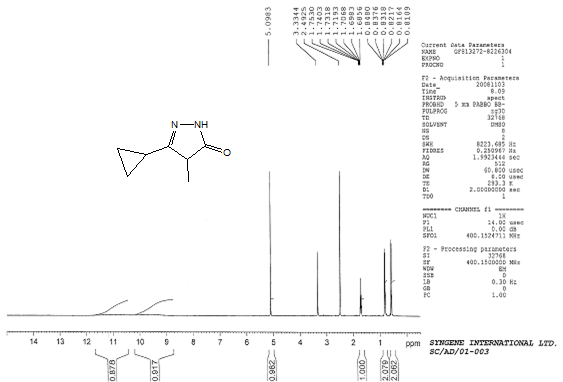
**

Fig.44. ^1^H NMR spectrum of 3-Cyclopropyl-1*H*-pyrazol-5(4*H*)-one **(25)**


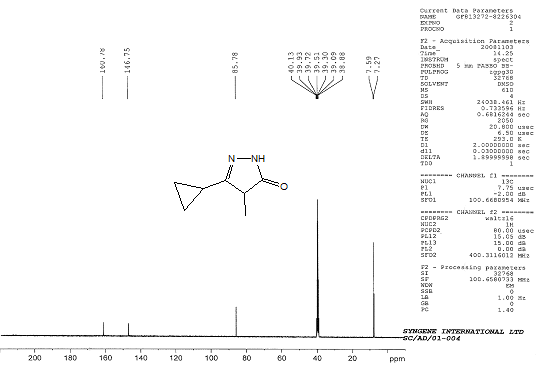


Fig.45 ^13^C NMR spectrum of 3-Cyclopropyl-1*H*-pyrazol-5(4*H*)-one **(25)**


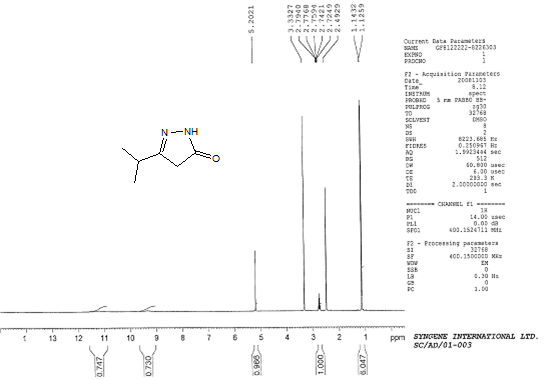


Fig. 46 ^1^H NMR spectrum of 3-*Iso*propyl-1*H*-pyrazol-5(4*H*)-one **(26)**


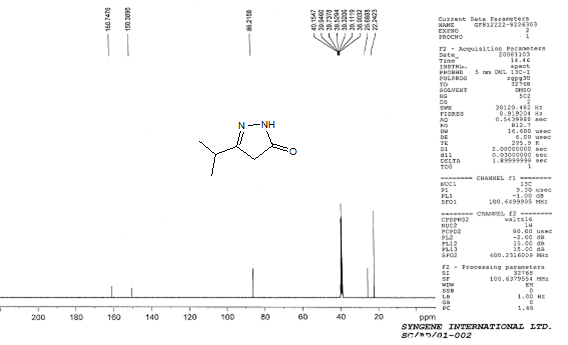


Fig.47 ^13^C NMR spectrum of 3-*Iso*propyl-1*H*-pyrazol-5(4*H*)-one **(26)**


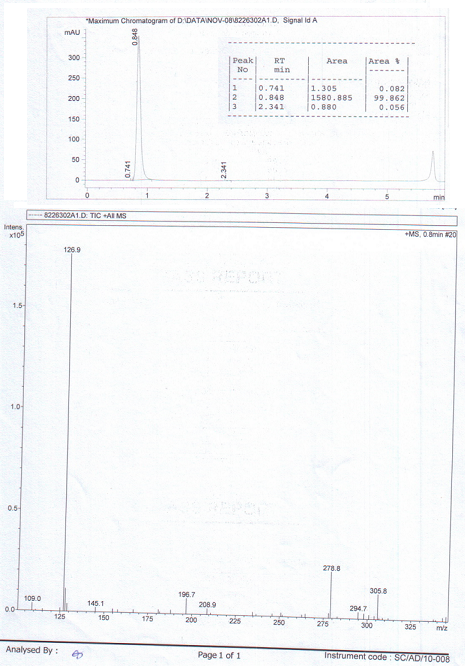


Fig.48 LC-MS spectrum of the compound **26**

Fig.49. ORTEP plot of 4-Methyl-3-phenyl-1*H*-pyrazol-3(4*H*)-one (**4** in **enol** form)

Fig.49. ORTEP plot of 4-Methyl-3-phenyl-1*H*-pyrazol-3(4*H*)-one (**4** in **keto** form)

ORTEP plot of 4,5,6,7,8,9-Hexahydro-2*H*-cycloocta[c]pyrazol-3(3a*H*)-one (**7**)


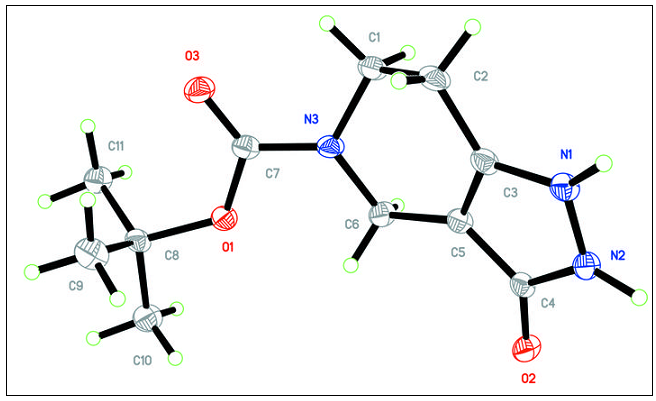


Fig.50. ORTEP plot of *Tert*-butyl 3-oxo-2,3,3a,4,6,7-hexahydropyrazolo[4,3-c]pyridine-5-carboxylate **(16).**

Fig. 51 ORTEP plot of 5-Ethyl-4-methyl-1*H*-pyrazol-3(2*H*)-one (**21**)

ORTEP Plot of 5-Ethyl-4-methyl-1*H*-pyrazol-3(2*H*)-ium salt (**21**)

ORTEP Plot of 4-Ethyl-3-phenyl-1*H*-pyrazol-5(4*H*)-one (**23**)

ORTEP Plot of 3-Cyclohexyl-4-methyl-1*H*-pyrazol-5(4*H*)-one (**24**)
